# Massed vs Intensive Outpatient Prolonged Exposure for Combat-Related Posttraumatic Stress Disorder: A Randomized Clinical Trial

**DOI:** 10.1001/jamanetworkopen.2022.49422

**Published:** 2023-01-05

**Authors:** Alan L. Peterson, Tabatha H. Blount, Edna B. Foa, Lily A. Brown, Carmen P. McLean, Jim Mintz, Richard P. Schobitz, Bryann R. DeBeer, Joseph Mignogna, Brooke A. Fina, Wyatt R. Evans, Samantha Synett, Brittany N. Hall-Clark, Timothy O. Rentz, Christian Schrader, Jeffrey S. Yarvis, Katherine A. Dondanville, Hunter Hansen, Vanessa M. Jacoby, Jose Lara-Ruiz, Casey L. Straud, Willie J. Hale, Dhiya Shah, Lauren M. Koch, Kelsi M. Gerwell, Stacey Young-McCaughan, Brett T. Litz, Eric C. Meyer, Abby E. Blankenship, Douglas E. Williamson, John D. Roache, Martin A. Javors, Allah-Fard M. Sharrieff, Barbara L. Niles, Terence M. Keane

**Affiliations:** 1 Department of Psychiatry and Behavioral Sciences, University of Texas Health Science Center at San Antonio, San Antonio; 2 Research and Development Service, South Texas Veterans Health Care System, San Antonio; 3 Department of Psychology, University of Texas at San Antonio, San Antonio; 4 Center for the Treatment and Study of Anxiety, Department of Psychiatry, University of Pennsylvania, Philadelphia; 5 Dissemination and Training Division, National Center for PTSD, VA Palo Alto Health Care System, Menlo Park, California; 6 Department of Psychiatry and Behavioral Sciences, Stanford University, Stanford, California; 7 Department of Population Health Sciences, University of Texas Health Science Center at San Antonio, San Antonio; 8 Department of Behavioral Health, Brooke Army Medical Center, Joint Base San Antonio-Fort Sam Houston, Texas; 9 VISN-17 Center of Excellence for Returning War Veterans, Central Texas Veterans Health Care System, Waco; 10 Rocky Mountain Mental Illness Research Education and Clinical Center for Suicide Prevention, Department of Veterans Affairs, Aurora, Colorado; 11 Department of Behavioral Health, Carl R. Darnall Army Medical Center, Fort Hood, Texas; 12 School of Social Work, Tulane University, New Orleans, Louisiana; 13 Department of Psychiatry, School of Medicine, Boston University, Boston, Massachusetts; 14 Massachusetts Veterans Epidemiology Research and Information Center, VA Boston Healthcare System, Boston; 15 Department of Psychological and Brain Sciences, School of Arts and Sciences, Boston University, Boston, Massachusetts; 16 Department of Rehabilitation Science and Technology, University of Pittsburgh, Pittsburgh, Pennsylvania; 17 Department of Psychiatry and Behavioral Sciences, Duke University, Durham, North Carolina; 18 Durham VA Health Care System, Durham, North Carolina; 19 Department of Homeland Security, Miami, Florida; 20 Behavioral Science Division, National Center for PTSD, VA Boston Healthcare System, Boston, Massachusetts

## Abstract

**Importance:**

Improved, efficient, and acceptable treatments are needed for combat-related posttraumatic stress disorder (PTSD).

**Objective:**

To determine the efficacy of 2 compressed prolonged exposure (PE) therapy outpatient treatments for combat-related PTSD.

**Design, Setting, and Participants:**

This randomized clinical trial was conducted among military personnel and veterans at 4 sites in Texas from 2017 to 2019. Assessors were blinded to conditions. Data were analyzed from November 2020 to October 2022.

**Interventions:**

The interventions were massed-PE, which included 15 therapy sessions of 90 minutes each over 3 weeks, vs intensive outpatient program PE (IOP-PE), which included 15 full-day therapy sessions over 3 weeks with 8 treatment augmentations. The IOP-PE intervention was hypothesized to be superior to massed-PE.

**Main Outcomes and Measures:**

Coprimary outcomes included the Clinician-Administered PTSD Scale for *Diagnostic and Statistical Manual of Mental Disorders* (Fifth Edition) (*DSM-5*) (CAPS-5) and the PTSD Checklist for *DSM-5* (PCL-5) administered at baseline and posttreatment follow-ups. Measures ranged from 0 to 80, with higher scores indicating greater severity. Diagnostic remission and reliable change were secondary outcomes.

**Results:**

Among 319 military personnel and veterans screened, 234 were randomized (mean [SD] age, 39.20 [7.72] years; 182 [78%] male participants), with 117 participants randomized to IOP-PE and 117 participants randomized to massed-PE. A total of 61 participants (26%) were African American, 58 participants (25%) were Hispanic, and 102 participants (44%) were White; 151 participants (65%) were married. Linear mixed-effects models found that CAPS-5 scores decreased in both treatment groups at the 1-month follow-up (IOP-PE: mean difference, −13.85 [95% CI, −16.47 to −11.23]; *P* < .001; massed-PE: mean difference, −14.13 [95% CI, −16.63 to −11.62]; *P* < .001). CAPS-5 change scores differed from 1- to 6-month follow-ups (mean difference, 4.44 [95% CI, 0.89 to 8.01]; *P* = .02). PTSD symptoms increased in massed-PE participants during follow-up (mean difference, 3.21 [95% CI, 0.65 to 5.77]; *P* = .01), whereas IOP-PE participants maintained treatment gains (mean difference, 1.23 [95% CI, −3.72 to 1.27]; *P* = .33). PCL-5 scores decreased in both groups from baseline to 1-month follow-up (IOP-PE: mean difference, −21.81 [95% CI, −25.57 to −18.04]; *P* < .001; massed-PE: mean difference, −19.96 [95% CI, −23.56 to −16.35]; *P* < .001) and were maintained at 6 months (IOP-PE: mean change, −0.21 [95% CI, −3.47 to 3.06]; *P* = .90; massed-PE: mean change, 3.02 [95% CI, −0.36 to 6.40]; *P* = .08). Both groups had notable PTSD diagnostic remission at posttreatment (IOP-PE: 48% [95% CI, 36% to 61%] of participants; massed-PE: 62% [95% CI, 51% to 73%] of participants), which was maintained at 6 months (IOP-PE: 53% [95% CI, 40% to 66%] of participants; massed-PE: 52% [95% CI, 38% to 66%] of participants). Most participants demonstrated reliable change on the CAPS-5 (61% [95% CI, 52% to 69%] of participants) and the PCL-5 (74% [95% CI, 66% to 81%] of participants) at the 1-month follow-up.

**Conclusions and Relevance:**

These findings suggest that PE can be adapted into compressed treatment formats that effectively reduce PTSD symptoms.

**Trial Registration:**

ClinicalTrials.gov Identifier: NCT03529435

## Key Points

**Question:**

Can an intensive outpatient format of prolonged exposure therapy result in larger PTSD reductions than massed outpatient format prolonged exposure therapy?

**Findings:**

In this randomized clinical trial involving 234 military personnel and veterans treated with massed or intensive outpatient formats of prolonged exposure therapy, 61% achieved clinically significant reductions in clinician-assessed PTSD symptoms and 74% had self-reported PTSD symptom reductions at the 1-month follow-up assessment. There were no significant differences between the treatment groups, and in both, more than 50% of participants maintained PTSD diagnostic remission at the 6-month follow-up.

**Meaning:**

These findings suggest that both massed and intensive outpatient forms of prolonged exposure therapy are fast and effective for combat-related PTSD.

## Teaser

This randomized clinical trial assesses the efficacy of 2 prolonged exposure outpatient treatments for combat-related posttraumatic stress disorder.

## Introduction

The military conflicts in Iraq and Afghanistan over the past 2 decades have been the longest military operations in US history. Almost 3 million military personnel have deployed, resulting in more than 7000 deaths and 53 000 service members wounded.^1^ Posttraumatic stress disorder (PTSD) is a signature wound of war and the most common psychological health condition associated with combat deployments.^2,3,4^ Between 4% and 17% of US military veterans from the era after the September 11, 2001, terrorist attacks (hereafter, *9/11*; approximately 120 000 to 510 000 individuals) may have combat-related PTSD.^1,3,5^ The individual and societal costs of PTSD are substantial and have been well documented.^6,7,8,9,10^

When the wars in Iraq and Afghanistan began, there were no published PTSD clinical trials in active-duty US military personnel to inform clinicians treating this population.^11^ Over 2 decades, more than 20 clinical trials have evaluated various PTSD treatments.^12^ One treatment that has been extensively investigated is prolonged exposure (PE) therapy.^13^ The first randomized clinical trial (RCT) to evaluate the efficacy of PE for combat-related PTSD in active-duty US military personnel^14,15^ compared the standard treatment format of PE (ie, spaced PE, which includes 10 weekly 90-minute sessions) to a massed treatment format (massed-PE, which includes 10 daily 90-minute sessions over 2 weeks). Daily PE was thought to be a more efficient, feasible, and acceptable treatment approach for active-duty military. Both treatments resulted in significant reductions in clinician-administered (spaced-PE: *d* = 0.84; massed-PE: *d* = 1.04) and self-reported (spaced-PE: *d* = 1.13; massed-PE: *d* = 1.27) measures of PTSD symptoms that were maintained over a 6-month follow-up period. However, the massed-PE format reduced treatment dropouts by almost half (spaced-PE: 24.8% of participants; massed-PE: 13.6% of participants), suggesting improved efficiency, feasibility, and acceptability for military populations.

Although these initial results indicated that PE was effective for the treatment of combat-related PTSD, approximately 60% of participants continued to meet diagnostic criteria for PTSD at the 6-month follow-up. This suggested that further adaptations to the standard treatment protocols may be required to address unique aspects of combat-related PTSD.^16,17^ For example, although combat veterans are exposed to multiple types of traumas, standard PE focuses on the single most distressing trauma to facilitate generalization to less distressing memories.^18^ Adapting treatments to procedurally focus on multiple traumas may improve outcomes. Effective treatment of combat-related PTSD may also require more intensive treatment protocols. In theory, intensive outpatient programs (IOPs) and residential inpatient treatment programs are the highest level of care for PTSD. However, despite the proliferation of IOPs^19,20,21,22^ and residential programs^23,24^ for PTSD, no previous RCTs have evaluated either form of these intensive treatment programs for US military personnel and veterans.

In 2022, the war in Ukraine raised international concerns regarding the risk of PTSD in military personnel and civilians.^25^ Additional research is required to extend current findings and to adapt PTSD treatment protocols to the needs of specific populations. The objective of this RCT was to compare 2 versions of compressed PTSD treatment protocols, massed-PE and IOP-PE, each delivered over a 3-week period, among US military personnel and veterans. It was hypothesized that both treatments would result in significant reductions in clinician-assessed and self-report PTSD symptoms but that the IOP-PE intervention would be superior to massed-PE.

## Methods

This RCT was approved by the institutional review board at the University of Texas Health Science Center at San Antonio. Participants provided written informed consent, completed a baseline screening to determine eligibility, and were randomly assigned to treatment condition by the project biostatistician using an SAS random allocation sequence program version 9.3 (SAS Institute) with block sizes of 2, 4, and 6. This report follows the Consolidated Standards of Reporting Trials (CONSORT) reporting guideline for randomized studies.

### Design and Sample

**Figure 1.**
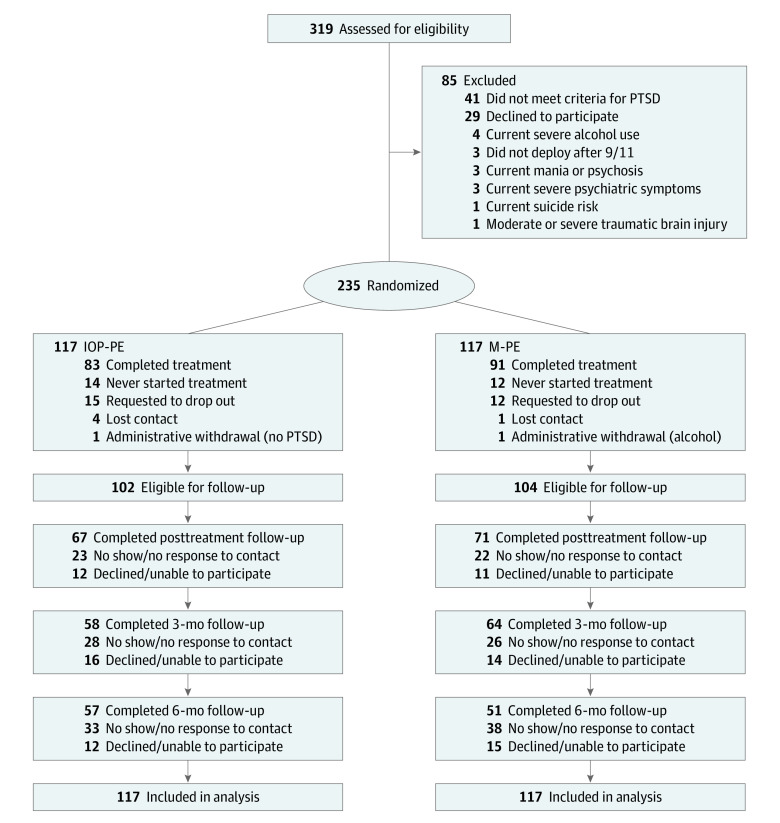
Participant Recruitment Flowchart IOP-PE indicates intensive outpatient program prolonged exposure; M-PE, massed prolonged exposure; PTSD, posttraumatic stress disorder.

Participants were recruited from 4 sites in South-Central Texas, including 2 US military treatment facilities and 2 US Department of Veterans Affairs (VA) facilities. Military personnel and veterans provided consent and were screened for the study beginning in November 2016. Eligible participants were randomized 1:1 to each arm (Figure 1). Follow-up data collection was completed in November 2019. Additional details about the research methods for this have been published elsewhere^26^ and are provided in the trial protocol and statistical analysis plan in Supplement 1.

### Inclusion and Exclusion Criteria

Participants were active duty service members or veterans who had deployed in support of post-9/11 US military combat operations. All participants had experienced at least 1 deployment-related criterion A event and met diagnostic criteria for PTSD.^6,27^ Participants were English-speaking, and those using psychotropic medications were receiving stable doses. Exclusion criteria included active symptoms that might interfere with treatment, such as mania, psychosis, substance use, suicidality, or psychiatric condition requiring immediate clinical attention.

### Treatment Conditions

Participants were randomly assigned to IOP-PE or massed-PE. PE^28,29^ served as the foundation for both treatment protocols. PE targets psychological mechanisms that maintain PTSD and includes a treatment rationale, psychoeducation about trauma, diaphragmatic breathing, in vivo exposure, imaginal exposure, and processing of the trauma.

#### Massed Prolonged Exposure

The massed-PE protocol was the active comparison condition. It included all the components of PE, but they were delivered in 15 daily 90-minute PE sessions over 3 consecutive weeks rather than once-weekly sessions.^26^

#### Intensive Outpatient Program Prolonged Exposure

The IOP-PE protocol also included 15 daily, 90-minute PE sessions delivered over 3 consecutive weeks, with 8 additional treatment adaptations.^26^ It was hypothesized that these enhancements would improve treatment outcomes by addressing the clinical issues that are germane to combat-related PTSD. The rationale for these treatment augmentations is included in eTable 1 in Supplement 2.

### Procedures

Treatment was provided by study therapists (T.H.B., B.A.F., W.R.E., S.S., B.N.H.-C., H.H., V.M.J., C.L.S., D.S., L.M.K., and K.M.G.) with at least a master’s degree in clinical psychology or social work and who had extensive training in PE. Protocol adherence was assessed during weekly study team meetings. Participants completed assessments during treatment (following treatment days 5, 10, and 14) and at 1, 3, and 6 months after treatment. All assessments were administered by master’s- or doctoral-level independent evaluators who were blinded to treatment condition.^26^

### Measures

Coprimary outcomes included the Clinician-Administered PTSD Scale for *Diagnostic and Statistical Manual of Mental Disorders* (Fifth Edition) (*DSM-5*)^6^ (CAPS-5)^27,30,31^ and the PTSD Checklist for *DSM-5* (PCL-5)^32,33,34^ administered at baseline and posttreatment follow-ups. Diagnostic remission and reliable change were secondary outcomes. Detailed descriptions of these measures have been published elsewhere^26^ and are included in eTable 2 in Supplement 2.

#### Demographics and Military Service Characteristics Form

The Demographics and Military Service Characteristics Form is a self-reported measure of standard demographic characteristics and information related to military service. Race and ethnicity data were collected from self-report on this form and classified as African American, Hispanic, non-Hispanic White, and other, including individuals who identified as multiracial. Race and ethnicity were assessed to provide the demographic characteristics of the participants.

#### Clinician Administered PTSD Scale for *DSM-5*

The CAPS-5^27,30,31^ is a structured diagnostic interview assessing for PTSD. It was administered at baseline and the 1-, 3-, and 6-month follow-ups. Scores range from 0 to 80, with higher score indicating more severe PTSD.

#### PTSD Checklist for *DSM-5*

The PCL-5^32,33,34^ is a 20-item self-reported measure of PTSD symptoms. It was administered at baseline, weekly during treatment (interim assessments 1, 2, and 3), and at the 1-, 3-, and 6-month follow-up assessments. Scores range from 0 to 80, with higher score indicating more severe PTSD.

#### Sheehan Disability Scale

The Sheehan Disability Scale (SDS)^35,36,37,38,39^ is a 3-item self-reported measure of functional impairment due to mental health difficulties. It was administered at baseline and at the 1-, 3-, and 6-month follow-ups. Scores range from 0 to 30, with higher score indicating greater impairment.

#### Brief Inventory of Psychosocial Functioning

The Brief Inventory of Psychosocial Functioning (B-IPF)^40,41^ is a 7-item, self-reported measure of psychosocial functioning. It was administered at baseline and at the 1-, 3-, and 6-month follow-ups. Scores range from 0 to 6 with higher score indicating more struggles with functioning.

#### Adverse Event Monitoring

Participants were formally assessed once weekly during treatment for adverse events (AEs)^42^ they experienced in the past week. Adjudication of AEs was conducted during weekly study team meetings.

### Power Analysis

A power analysis was calculated using PASS power software version 11.0 (NCSS Statistical Software) to test the hypothesis that the IOP-PE would be superior to massed-PE on PTSD outcomes. The primary hypotheses were tests of change-score differences on PTSD symptom severity as measured by the CAPS-5 and PCL-5 at 2 time points (baseline to 1-month follow-up and 1-month follow-up to 6-month follow-up). An estimated statistical power of 80% was determined assuming standardized mean slope difference (Cohen *d*) of 0.40, an *r* = 0.50 within-participant measurements, 100 participants equally allocated to each treatment group, and a 2-tailed, unadjusted *P* = .05. Power calculations also yielded the desired statistical power of 80% when the proportional difference in PTSD diagnostic remission on the CAPS-5 was approximately 20% with 100 participants and a 2-tailed, unadjusted *P* = .05.

### Statistical Analysis

Intent-to-treat statistical analyses were used. Hypothesis tests were 2-tailed and unadjusted at *P* = .05. We used SPSS statistical software version 27 (IBM) for all analyses. Prior to statistical analyses, the data were inspected for problems, such as unusual distributions, the desirability of transformations, missing data, and outliers. Missing item-level data were minimal (<1%).

Linear mixed-effects models with repeated measures were used to evaluate continuous outcomes on the CAPS-5, PCL-5, SDS, and B-IPF with fixed main effects of group, time, and the group × time interaction effect. Piecewise pairwise comparisons were used to evaluate PTSD symptom reduction differences between the treatment groups from baseline to the 1-month follow-up and from the 1-month to 6-month follow-ups. Generalized linear models were used to evaluate treatment group differences in the proportion of participants who demonstrated PTSD diagnostic remission on the CAPS-5 at the 1-, 3-, and 6-month follow-ups. As a supplement to PTSD symptom and diagnostic remission outcomes, the proportion of participants who demonstrated a reliable change on the CAPS-5 and PCL-5 was calculated according to the Reliable Change Index (RCI)^43^ following treatment. The RCI is based on the SE of measurement of the difference between individuals’ baseline and posttreatment scores and is a function of the instrument’s SD and reliability. The SD and Cronbach α at baseline were used to calculate the RCI in this study. These estimates resulted in cutoff of 10 on both the CAPS-5 and PCL-5 when using a 1-tailed 95% confidence limit, and pre-post improvements that would occur by chance 4.5% of the time if there were no real change on the CAPS-5 and 4.8% of the time if there were no real change on the PCL-5. Generalized linear models were also used to evaluate treatment group differences in the proportion of participants who had RCI at 1-, 3-, and 6-month follow-ups. Data were analyzed from November 2020 to October 2022.

## Results

### Participants

Among 319 military personnel and veterans screened, 234 were randomized (mean [SD] age, 39.2 [7.8] years; 182 [78%] men), with 117 participants randomized to IOP-PE and 117 participants randomized to mass-PE. The sample was culturally diverse, including 61 African American participants (26%), 58 Hispanic participants (25%), 102 White participants (44%), and 7 participants (3%) identifying as another race or ethnicity. A total of 151 participants (65%) were married (Table 1).

**Table 1.** Demographic Characteristics of Included Participants

Characteristic	Participants, No. (%)^a^		
	Total (N = 234)	IOP-PE (n = 117)	M-PE n = 117)
Age, mean (SD), y	39.20 (7.72)	39.37 (7.27)	39.03 (8.16)
Gender			
Women	52 (22)	24 (20)	28 (24)
Men	182 (78)	93 (80)	89 (76)
Married	151 (65)	80 (68)	71 (61)
Race and ethnicity			
African American	61 (26)	33 (29)	28 (24)
Hispanic	58 (25)	30 (27)	28 (24)
Non-Hispanic White	102 (44)	45 (40)	57 (50)
Other^b^	7 (3)	5 (4)	2 (2)
Education			
≤High school	14 (6)	9 (8)	5 (4)
Some college or an associate degree	144 (62)	74 (63)	34 (29)
≥Bachelor’s degree	76 (32)	70 (60)	42 (36)
Active duty^c^	152 (65)	77 (66)	75 (64)
Army	191 (82)	92 (79)	26 (85)
Enlisted rank	200 (86)	101 (86)	99 (85)
Time in military, mean (SD), mo	182 (83)	184 (77)	180 (88)
Typical duty			
Combat arms	81 (35)	47 (41)	34 (29)
Combat support	63 (27)	32 (28)	31 (27)
Combat service support	89 (38)	37 (32)	52 (44)
Deployments, No.			
1	69 (30)	29 (25)	40 (34)
2	70 (30)	34 (29)	36 (31)
3	48 (20)	26 (22)	22 (20)
≥4	46 (20)	27 (23)	19 (16)

Abbreviations: IOP-PE, intensive outpatient program prolonged exposure; M-PE, massed prolonged exposure.

^a^ Cell counts vary based on available data across variables.

^b^ Includes participants who identified as multiple races or ethnicities.

^c^ Seven participants in this category were serving in the Army Reserve or National Guard.

### Treatment Completion

A treatment completer was defined as a participant who completed all 15 treatment sessions or responded early (33 participants) after at least 12 treatment sessions with at least a 10-point reduction on the PCL-5 and participant and therapist agreement that early termination was appropriate. There was not a significant difference in treatment completion between groups (IOP-PE: 82 participants [70%]; massed-PE: 90 participants [77%]; *P* = .24). Protocol adherence was greater than 90% for both conditions.

### Changes in PTSD Symptom Severity

**Figure 2.**
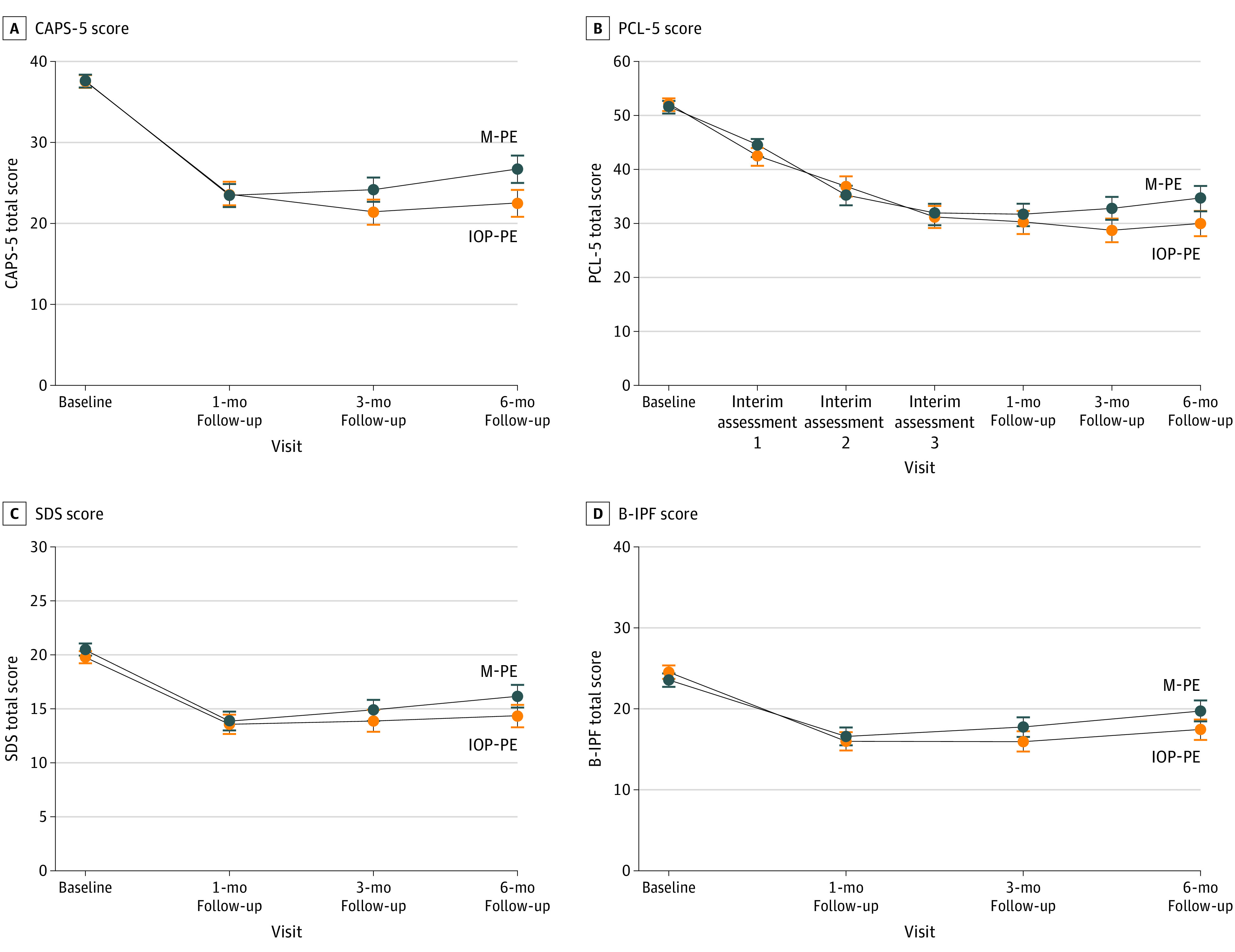
Changes in Assessment Scores by Treatment Group B-IPF indicates Brief Inventory of Psychosocial Functioning; CAPS-5, Clinician-Administered PTSD Scale for *Diagnostic and Statistical Manual of Mental Disorders* (*Fifth Edition*) (*DSM-5*); IOP-PE, intensive outpatient program prolonged exposure; M-PE, massed prolonged exposure; PCL-5, PTSD Checklist for *DSM-5*; SDS, Sheehan Disability Scale.

Estimated model means and SEs and key pairwise comparisons across symptom severity outcomes are presented in Table 2. There was a significant interaction effect found on the CAPS-5, suggesting that PTSD symptom reductions differed over time between the treatment groups (*F*_3,122.50_ = 2.78; *P* = .04). Deconstruction of the interaction effect indicated that CAPS-5 scores significantly decreased from baseline to the 1-month follow-up in both treatment groups (IOP-PE: mean change, −13.85 [95% CI, −16.47 to −11.23]; *P* < .001; *d* = −1.62; massed-PE: mean change, −14.13 [95% CI, −16.63 to −11.62]; *P* < .001; *d = *−1.65), but symptom reductions did not differ between treatment groups *(*mean difference*,* −0.27 [95% CI, −3.90 to 3.35]; *P* = .88; *d* = −0.03). There was a significant difference on PTSD symptom reductions between treatment groups from the 1-month follow-up to the 6-month follow-up (mean difference, 4.44 [95% CI, 0.86 to 8.01]; *P* = .01; *d = *0.52). Participants in the IOP-PE group maintained 1-month follow-up treatment gains at the 6-month follow-up (mean change, −1.23 [95% CI, −3.72 to 1.27]; *P* = .33; *d = *−0.14), whereas participants in the massed-PE showed a symptom increase on the CAPS-5 during this time (mean change, 3.21 [95% CI, 0.65 to 5.77]; *P* = .01; *d* = 0.37) (Figure 2A).

**Table 2.** PTSD Symptom Severity and Disability Impairment Outcomes^a^

Outcome	Score, mean (SD)				Key pairwise comparisons of treatment change score differences								
	Baseline	Follow-up, mo			Baseline vs 1-mo follow-up				1-mo vs 6-mo follow-ups			
		1	3	6	MD	*t*	*P* value	*d*	MD	*t*	*P* value	*d*
**CAPS-5**													
IOP-PE	37.56 (0.79)	23.70 (1.47)	21.42 (1.56)	22.48 (1.67)	−0.27	−0.15	.88	−0.03	4.44	2.46	.01	0.52
M-PE	37.59 (0.79)	23.46 (1.42)	24.17 (1.50)	26.67 (1.69)								
**PCL-5**													
IOP-PE	51.99 (1.17)	30.19 (2.15)	28.72 (2.19)	29.98 (2.34)	1.85	0.70	.48	0.15	3.23	1.36	.18	0.26
M-PE	51.53 (1.17)	31.57 (2.08)	32.79 (2.13)	34.60 (2.35)								
**SDS**													
IOP-PE	20.83 (0.58)	14.86 (0.95)	14.85 (1.02)	15.13 (1.12)	−0.41	−0.32	.75	−0.07	1.55	1.13	.26	0.26
M-PE	21.56 (0.57)	15.7 (0.98)	15.57 (0.99)	17.68 (1.13)								
**B-IPF**													
IOP-PE	24.53 (0.83)	15.98 (1.13)	15.97 (1.25)	17.42 (1.26)	1.59	1.04	.30	0.18	1.71	1.17	.25	0.19
M-PE	23.54 (0.83)	16.59 (1.10)	17.75 (1.20)	19.73 (1.29)								

Abbreviations: B-IPF, Brief Inventory of Psychosocial Functioning; CAPS-5, Clinician Administered PTSD Scale *Diagnostic and Statistical Manual of Mental Disorders* (*Fifth Edition*) (*DSM-5)*; IOP-PE, intensive outpatient prolonged exposure; MD, between-group mean difference in change; M-PE, massed prolonged exposure; PCL-5, PTSD Checklist *DSM-5*; SDS, Sheehan Disability Scale.

^a^ Both treatment groups demonstrated significant reductions on all outcomes from baseline to follow-up time points.

On the PCL-5, there was not a significant interaction effect observed, which suggests that PTSD symptom reductions did not significantly differ between treatment groups (Figure 2B). There was a main effect of time, indicating that both treatments demonstrated significant PTSD reductions from baseline to the 1-month follow-up (IOP-PE: mean change, −21.81 [95% CI, −25.57 to −18.04]; *P* < .001; *d* = −1.72; massed-PE: mean change, −19.96 [95% CI, −23.56 to −16.35]; *P* < .001; *d = *−1.58) that were maintained at the 6-month follow-up (IOP-PE: mean change, −0.21 [95% CI, −3.47 to 3.06]; *P* = .90; *d* = −0.02; massed-PE: mean change, 3.02 [95% CI, −0.36 to 6.40]; *P* = .08; *d* = 0.24).

### PTSD Diagnostic Remission

**Figure 3.**
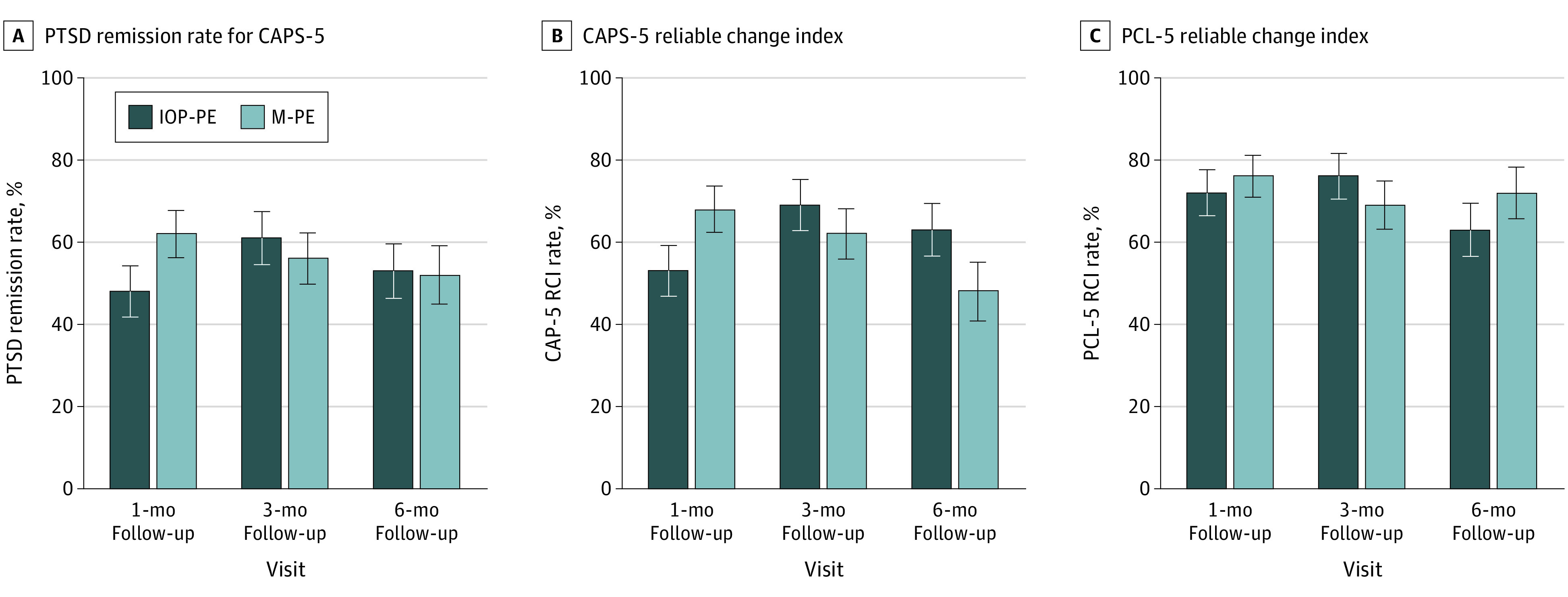
Posttraumatic Stress Disorder (PTSD) Diagnostic Remission Rate and Reliable Change Index (RCI) by Treatment Group CAPS-5 indicates Clinician-Administered PTSD Scale for *Diagnostic and Statistical Manual of Mental Disorders* (*Fifth Edition*) (*DSM-5*); IOP-PE, intensive outpatient program prolonged exposure; M-PE, massed prolonged exposure; and PCL-5, PTSD Checklist for *DSM-5*.

Both treatments had notable loss of PTSD diagnosis at 1-, 3-, and 6-month follow-ups (Figure 3A). At the 1-month follow-up, 48% (95% CI, 36% to 61%) of IOP-PE participants and 62% (95% CI, 51% to 73%) of massed-PE participants no longer met diagnostic criteria for PTSD on the CAPS-5. PTSD diagnostic remission was maintained in approximately half of the IOP-PE (53% [95% CI, 40% to 66%] of participants) and massed-PE (52% [95% CI, 38% to 66%] of participants) participants at the 6-month follow-up. PTSD diagnostic remission rates were not significantly different at the 1-, 3-, or 6-month follow-ups.

### Reliable Change Index

At the 1-month follow-up, 61% (95% CI, 52% to 69%) of participants in both treatment groups had change scores that exceeded the RCI threshold on the CAPS-5 (IOP-PE: 53% [95% CI, 41% to 65%] of participants; massed-PE: 68% [95% CI, 57% to 78%] of participants); (Figure 3B), and 74% (95% CI, 66% to 81%) of all participants had change scores exceeding the RCI threshold on the PCL-5 (IOP-PE: 72% [95% CI, 61% to 83%] of participants; massed-PE: 76% [95% CI, 66% to 86%] of participants). (Figure 3C). Similarly, participants in both treatments demonstrated high rates of reliable change at the 6-month follow-up on the CAPS-5 (IOP-PE: 63% [95% CI, 51% to 76%] of participants; massed-PE: 48% [95% CI, 34% to 62%] of participants) and the PCL-5 (IOP-PE: 63% [95% CI, 50% to 75%] of participants; massed-PE: 72% [95% CI, 60% to 84%] of participants). The proportion of participants with RCI was not significantly different on either measure at the 1-, 3-, or 6-month follow-ups.

### Disability and Psychosocial Functioning Outcomes

There was not a significant interaction effect on the SDS or B-IPF, indicating that change scores did not significantly differ between treatment groups (Figure 2C and D). There was a main effect of time on the SDS and B-IPF. SDS total scores significantly decreased in both treatment groups from baseline to the 1-month follow-up (IOP-PE: mean change, −6.21 [95% CI, −8.05 to −4.36]; *P* < .001; *d* = −1.02; massed-PE: mean change, −6.62 [95% CI, −8.42 to −4.83]; *P* < .001; *d* = −1.09). SDS improvements were maintained at the 6-month follow-up in the IOP-PE treatment group (mean change, 0.78 [95% CI, −1.15 to 2.64]; *P* = .44; *d* = 0.12) and meaningfully increased in the massed-PE group (mean change, 2.29 [95% CI, 0.35 to 4.24]; *P* = .02; *d* = 0.38). However, there was not a significant difference in score change between treatment groups on the SDS from 1- to 6-month follow-up (Table 2). B-IPF total scores also significantly decreased in both treatment groups from baseline to the 1-month follow-up (IOP-PE: mean change, −8.54 [95% CI, −10.70 to −6.38]; *P* < .001; *d* = −0.96; massed-PE: mean change, −6.95 [95% CI, −9.06, −4.85]; *P* < .001; *d* = −0.78). Changes on the B-IPF total score were maintained at the 6-month follow-up with IOP-PE (mean change_,_ 1.43 [95% CI, −0.58 to 3.45]; *P* = .16; *d* = 0.16) and significantly increased with massed-PE (mean change_,_ 3.14 [95% CI, 1.05 to 5.24]; *P* = .004; *d* = 0.35). There was not a significant difference in change scores on the B-IPF between treatment groups from the 1-month to 6-month follow-ups (Table 2).

### Adverse Events Reported

A total of 49 participants reported a total of 154 AEs (eTable 3 in Supplement 2). There were no significant differences in AEs associated with IOP-PE (21% [95% CI, 15% to 30%] of participants) and massed-PE (21% [95% CI, 15% to 30%] of participants). There were 2 serious AEs in the IOP-PE group that were unrelated to treatment (accident and pneumonia) and 1 hospitalization for suicide risk of unknown or possible relationship to massed-PE after 2 weeks of treatment.

## Discussion

This RCT found that 2 PE protocols compressed to 3 weeks each demonstrated before-to-after treatment improvements in post-9/11 US military service members and veterans with PTSD. These findings provide strong support that combat-related PTSD can be effectively treated. Significant posttreatment reductions in clinician-rated and self-reported PTSD symptoms were seen across both therapies, and these changes were statistically significant and clinically meaningful. Approximately two-thirds of all participants reported clinically meaningful improvements in their clinician-diagnosed and self-reported PTSD symptoms, and more than half no longer qualified for a PTSD diagnosis. Notable posttreatment reductions also occurred in disability and functional impairment across both treatments. Compared with the previous 2-week massed-PE treatment protocol,^14^ the greater levels of improvement seen in the current 3-week protocol suggest that some post-9/11 service members with combat-related PTSD may require more treatment sessions to obtain such substantial reductions in symptoms.^14,44^ Effective treatments are needed to mitigate the long-term negative consequences of PTSD in military service members, veterans, and civilians, including psychological casualties from the war in Ukraine. Previous research has shown that PE retains its effectiveness when used in a compressed 2-week PE protocol.^14^

### Limitations

This study had several limitations. Most of the active duty military and veteran participants were recruited from Texas and may not represent the entire US population of service members and veterans. Because of ethical concerns about not treating all participants, this trial compared 2 compressed formats of PE delivery and did not include a waiting list control, placebo, or an alternative inactive treatment. Therefore, additional research is needed to determine the comparative efficacy of compressed PE and to match participants to treatments to help attain even greater treatment efficacy. In addition, daily compressed PTSD treatments delivered over 2 to 3 weeks are not feasible for all individuals. However, the results of this study are consistent with other research showing that compressed treatment formats result in more patients completing treatment, reduced treatment dropouts, and overall improved outcomes.^14,44,45,46,47^ Although both compressed PE treatments in this study yielded similar results, the IOP-PE intervention was undoubtedly more expensive and required more participant and clinician effort. It is unclear if the additional costs are warranted for the better maintenance of the treatment benefits with the IOP-PE treatment.

## Conclusions

In this RCT, PE delivered in compressed formats and adapted to the military context resulted in significant, meaningful, and lasting improvements in PTSD, disability, and functional impairment for most participants. Given the previously identified limitations of PE for military-related PTSD,^48^ the results of this study provide important new evidence that combat-related PTSD can be effectively treated. The compressed treatment formats evaluated in this study also provide a potential for new alternative modes of therapy using combined treatments, medications, and devices.

## Notes

Supplement 1.
Trial Protocol and Statistical Analysis Plan

Supplement 2. **eTable 1.** Intensive Outpatient Program Prolonged Exposure (IOP-PE) Treatment Augmentations **eTable 2.** Description of Study Measures **eTable 3.** Summary of Adverse Events (AEs) Reported During Treatment
eReferences.

Supplement 3.
Data Sharing Statement
