# Supplementary material for: Massed vs Intensive Outpatient Prolonged Exposure for Combat-Related Posttraumatic Stress Disorder: A Randomized Clinical Trial
Source: JAMA Netw Open. 2023 Jan 5;6(1):e2249422. doi: 10.1001/jamanetworkopen.2022.49422 (PMC9856757; doi:10.1001/jamanetworkopen.2022.49422)
Supplement: Supplement 1. — Trial Protocol and Statistical Analysis Plan [file jamanetwopen-e2249422-s001.pdf]

HUMAN SUBJECTS RESEARCH PROTOCOL

1. PROTOCOL TITLE: Project Remission: Maximizing Outcomes with Intensive Treatments for Combat-Related PTSD

2. ABSTRACT

With an estimated 200,000 – 600,000 post-9/11 service members and veterans suffering from combat-related posttraumatic stress disorder (PTSD; 7-20% of the 3 million who have deployed), there is an urgent need for accessible therapies that are highly effective in quickly treating PTSD to remission, enabling service members to stay fit for active duty and veterans to integrate well into civilian life. However, recent studies indicate that post-9/11 veterans and active duty service members do not derived the same degree of benefit from evidence-based behavioral treatments (e.g., Prolonged Exposure; Cognitive Processing Therapy) as their civilian counterparts. This study examines whether intensive Prolonged Exposure protocols can improve on treatment outcomes for post-911 service members and veterans with combat-related PTSD.

The purpose of this randomized controlled trial is to compare the efficacy of 15 sessions of Massed-Prolonged Exposure (Massed-PE) delivered over 3-weeks to 15 days of an Intensive Outpatient Prolonged Exposure protocol (IOP-PE) delivered over 3-weeks. In comparison with standard outpatient PE, Massed-PE has been modified to include additional sessions and release from duty. IOP-PE has been further augmented and includes the following modifications: 1) Clinic-based completion of daily homework assignments; 2. Brief therapist feedback sessions; 3. Enhanced social support; 4. Focus on 3 traumas (versus single trauma event); 5. Graduated imaginal exposure; and 6. Posttreatment booster sessions. Up to 400 post-9/11 active duty military and veterans will be consented to obtain data from 250 for analysis. The main outcomes of the study include PTSD diagnoses and symptom severity along with psychosocial functioning and level of disability. Participants will be assessed at pre-treatment, while in-treatment, and 1-, 3- and 6-months after treatment completion.

3. OBJECTIVES/SPECIFIC AIMS/RESEARCH QUESTIONS

Objective 1: To conduct a two-group randomized clinical trial to evaluate the efficacy of Massed-Prolonged Exposure (Massed-PE) versus Intensive Outpatient Program Prolonged Exposure (IOP-PE) for the treatment of combat-related PTSD in active duty service members and veterans who deployed post-9/11.

Hypothesis 1: The IOP-PE treatment will result in larger reductions than the Massed-PE treatment in clinician-assessed PTSD symptoms on the Clinician Administered PTSD Scale-DSM-5 (CAPS-5) and self-reported PTSD symptoms on the PTSD Checklist-DSM-5 (PCL-5) at the posttreatment, 3-month, and 6-month follow-up points.

Hypothesis 2: The IOP-PE treatment will result in a larger percentage of participants who no longer meet clinician-assessed PTSD on the CAPS-5 than the Massed-PE treatment at the posttreatment, 3-month, and 6-month follow-up points.

Objective 2: To evaluate changes in disability and functional outcomes after Massed-PE and IOP-PE.

Hypothesis 3: The IOP-PE treatment will result in greater improvements than the Massed-PE treatment in functional outcomes on the Sheehan Disability Scale and the Brief Inventory of Psychosocial Functioning at the posttreatment, 3-month, and 6-month follow-up points.

4. MILITARY RELEVANCE: The Departments of Defense (DoD) and Veterans Affairs (VA) have reached a critical juncture regarding the treatment of combat-related posttraumatic stress disorder (PTSD) in active duty service members and veterans. Over the past 14 years, almost 3 million U.S. military personnel have deployed to Afghanistan, Iraq, and other locations in the Middle East, and it is estimated that 7-20% of service members and veterans have combat-related PTSD (Institute of Medicine, 2014; Richardson et al, 2010). Between 2002-2011, there was a significant increase in the overall U.S. military force strength (i.e., total number on active duty) in order to support two simultaneous military conflicts in Iraq and Afghanistan. With the official end of Operations Iraqi Freedom (OIF) in 2010, New Dawn (OND) in 2011, and Enduring Freedom (OEF) in 2014, a significant force reduction was initiated in 2011 for all branches of the U.S. military. The U.S. Army, for example, has been tasked to reduce the overall active duty force strength by approximately 120,000 Soldiers between 2012 and 2018 (Tan, 2015). With a significant force reduction in progress, active duty service members must be of the highest level of physical and psychological fitness in order to remain on active duty. A simple option is to

discharge service members with PTSD. However, service members with the most combat experience are not only at the highest risk for developing PTSD (Hoge et al., 2006; Sundin et al., 2014); they also possess the most critical skills, abilities, and knowledge to lead the U.S. military into the future. Therefore, it is in the best interest of the military to provide opportunities for service members with PTSD to receive highly efficacious treatments that enable them to become fully fit for worldwide duty, including possible future deployments. Because those who cannot be treated into remission (i.e., achieve significant reductions in PTSD symptoms that are below PTSD diagnostic thresholds) are at risk for discharge from active duty, treatments that are both highly efficacious for combat-related PTSD and accessible to active duty military personnel are urgently needed. If combat-related PTSD can be treated into remission for the majority of service members, it will also help de-stigmatize PTSD for the estimated 400,000 OEF/OIF/OND veterans with PTSD who have become eligible for VA health care since 2002 (Department of Veteran Affairs, 2015a; 2015b). Highly potent treatments for veterans are also imperative so they can be provided with the greatest opportunity for successful reintegration into civilian life with limited disability and functional impairment.

5. BACKGROUND AND SIGNIFICANCE. Trauma-focused treatments such as PE and Cognitive Processing Therapy (CPT) are the most frequently studied treatments for combat-related PTSD in veterans (Steenkamp et al., 2015). PE and CPT outperform waitlist and treatment-as-usual control conditions and demonstrate large posttreatment effect sizes (Cohen's d range: 0.78-1.10). However, as compared to PTSD in civilians, the treatment of combat-related PTSD in veterans has proven to be less successful. Whereas the majority of civilians reach good end state functioning following trauma-focused therapies (e.g., Resick et al., 2002), only about 40-50% of veterans with combat-related PTSD achieve significant reductions in PTSD symptoms and no longer meet the diagnostic threshold for PTSD at the end of treatment (Steenkamp et al., 2015). It has been hypothesized that treating combat-related PTSD in service members soon after trauma exposure and while they are still on active duty might result in better outcomes than previously found in studies of veterans (Peterson, Luethcke et al., 2011). The younger age, shorter duration of symptoms, desire to be treated into remission to remain on active duty, and stronger unit cohesion/social support (compared to Vietnam veterans, for example) are factors hypothesized to allow service members to achieve PTSD outcomes similar to those in civilian populations. Recently completed clinical trials by Drs. Edna Foa, Patricia Resick, and Jeffrey Cigrang as part of the STRONG STAR Consortium provide the strongest scientific evidence to date that combat-related PTSD can be successfully treated in active duty military personnel (Cigrang et al., 2011, 2015; Foa et al., 2015; Resick et al., 2015a, 2015b). Unfortunately, the initial results of these studies appear to be similar to those in veteran populations. This suggests that combat-related PTSD is uniquely different from PTSD in civilians and that treatments are needed that expand and augment established evidence-based treatments in order to address the distinctive elements of combat-related traumas.

PRELIMINARY EVIDENCE. Prolonged Exposure (PE) is the PTSD treatment most extensively evaluated in clinical trials with civilians and veterans, and there is strong scientific evidence to support its efficacy (Bisson, et al., 2007; Blount et al., 2013; Cigrang et al., 2005, 2011, 2015; Foa et al., 2015; Peterson, Foa et al., 2011; Peterson, Luethcke et al., 2011; Powers et al., 2010). The results of the numerous PE studies with a variety of populations led to the evaluation of PE in the largest randomized clinical trial (RCT) to date (N = 370) to evaluate the treatment of combat-related PTSD in an active duty military population. The study titled "Prolonged Exposure for PTSD among OEF/OIF Personnel: Massed vs. Spaced Trials" (PI: Edna Foa, PhD) was funded in 2008 by the DoD as part of the STRONG STAR Consortium. The study includes four treatment arms: (1) Spaced-PE; (n = 110; ten 90-minute weekly PE sessions); (2) Present Centered Therapy (n = 110; ten 90-minute weekly PCT sessions); (3) Massed-PE (n = 110; ten 90-minute daily PE sessions on weekdays over 2 weeks); and (4) Minimal Contact Control (n = 40; weekly contact over 4 weeks to provide a posttreatment comparison group for M-PE). An analysis was conducted of the first 75 participants randomized to Massed-PE versus the 40 participants randomized to the Minimal Contact Control (Foa et al., 2015). The results indicated that reductions in interviewer-assessed PTSD severity were significantly greater in the Massed-PE group than the Minimal Contact Control, and a larger proportion of Massed-PE participants lost their PTSD diagnosis (46%) as compared to the Minimal Contact Control group (23%). Similar results were obtained in a preliminary review of the initial 1-month posttreatment outcomes of the full RCT, indicating that the outcomes of the Massed-PE and Spaced-PE groups were nearly identical. This provides the first data to date to indicate that PE delivered in a massed format (i.e., ten 90-minute PE sessions delivered on weekdays over 2 weeks) will result in similar treatment outcomes to those of PE delivered in the standard spaced format (i.e., ten 90-minute PE sessions delivered over 8 weeks).

There are two primary limitations with the Massed-PE protocol used in the RCT at Fort Hood. First, service members were required to maintain their regular duty activities during the two-week treatment. Therefore, they may not have had sufficient time to complete their daily homework sessions (90 minutes to review session audiotape, 90 minutes to practice in vivo exercises, etc.). Second, only 10 treatment sessions were used with a focus on the worst trauma, which may have been an insufficient treatment dose for combat-related PTSD. The proposed study will augment the previous Massed-PE protocol using two intensified treatment formats.

6. RESEARCH DESIGN

The proposed randomized controlled trial will examine the efficacy of Massed-Prolonged Exposure (Massed-PE) versus Intensive Outpatient Program Prolonged Exposure (IOP-PE) for the treatment of combat-related PTSD in active duty service members and veterans who deployed post-9/11. Both treatments are active, therapist-delivered behavioral treatments. The design of the study is summarized in Figure 1.

The *independent variable* is the treatment condition (Massed-PE or IOP-PE) to which participants are assigned. Equal randomization between both groups will occur. For Hypotheses 1 and 2, the *primary dependent variables* include self-reported PTSD symptoms on the PTSD Checklist-DSM-5 (PCL-5) and PTSD diagnosis and severity as measured by Clinician-Administered PTSD Scale for DSM-5 (CAPS-5) administered by independent evaluators blind to study condition, respectively. For Hypothesis 3, the primary dependent variables include level of disability as measured by the Sheehan Disability Scale (Sheehan, 1983; Sheehan et al., 1996) and psychosocial functioning as measured by the Brief Inventory of Psychosocial Functioning (Marx, 2011). These measures will be administered at the pre-treatment, post-treatment, 3 month follow-up and 6-month follow up assessment periods.

Participants will be asked to complete the CAP Common Data Elements battery, which includes over 25 self-report measures, including: demographics and military history; personality; deployment stress, adversity, trauma; psychiatric symptoms and history; TBI; substance use; functional impairment; sources of support; sleep impairment; pain; relationship functioning; and treatment credibility expectancy as well as blood collection to use for evaluation of possible biomarkers of PTSD, response to PTSD treatment, and psychosocial functioning. This battery will be used to measure *secondary dependent variables* (i.e., changes in psychosocial functioning including psychiatric symptoms; substance use; functional impairment; sources of support; sleep impairment; pain; and relationship functioning) as well as key moderators and mediators (i.e., military history; personality; trauma history; TBI; treatment credibility and expectancy). A list of measures is provided below in section 7.3.

7. RESEARCH PLAN

7.1 Selection of Subjects

7.1.1. Subject Population. This study will consent and screen 400 active duty military personnel and veterans seeking behavioral health treatment for PTSD to randomize 250 for analysis. Both active duty and veterans will be recruited for treatment at one of four performance sites: the San Antonio Military Medical Center (SAMMC), located at Joint Base San Antonio (JBSA) - Fort Sam Houston, Texas; University of Texas Health Science Center, San Antonio, Texas; Carl. R. Darnall Army Medical Center at Fort Hood in Killeen, Texas; and Central Texas Veterans Health Care System, Doris Miller VA Medical Center, VISN 17 Center of Excellence for Research on Returning War Veterans, Waco, Texas. Enrollment will be competitive in an effort to meet the enrollment goals for the study.

7.1.2. Source of Research Material. All measures are being administered for research purposes. For a complete list of measures, see Section 7.3.

7.1.3. Inclusion and Exclusion Criteria.

Inclusion Criteria
1. Active duty military service member or veteran (age 18- 65 years) who deployed in support of combat operations post-9/11 seeking behavioral health treatment for PTSD.
2. PTSD diagnosis as assessed by Clinician-Administered Posttraumatic Stress Scale (CAPS-5).
3. Able to speak and read English (due to standardization of outcome measures)

Exclusion Criteria
1. Current manic episode or a psychotic symptoms requiring immediate stabilization or hospitalization (as determined by the bipolar and psychosis modules of the MINI).
2. Current and severe alcohol use warranting immediate intervention based on clinical judgment.
3. Evidence of a moderate or severe traumatic brain injury (as determined by the inability to comprehend the baseline screening questionnaires).
4. Current suicidal ideation severe enough to warrant immediate attention (as determined by the Depressive Symptoms Index-Suicidality Subscale and corroborated by a clinical risk assessment by a credentialed provider)

5. Other psychiatric disorders severe enough to warrant designation as the primary disorder as determined by clinician judgment.

Figure 1. Study Design Overview.

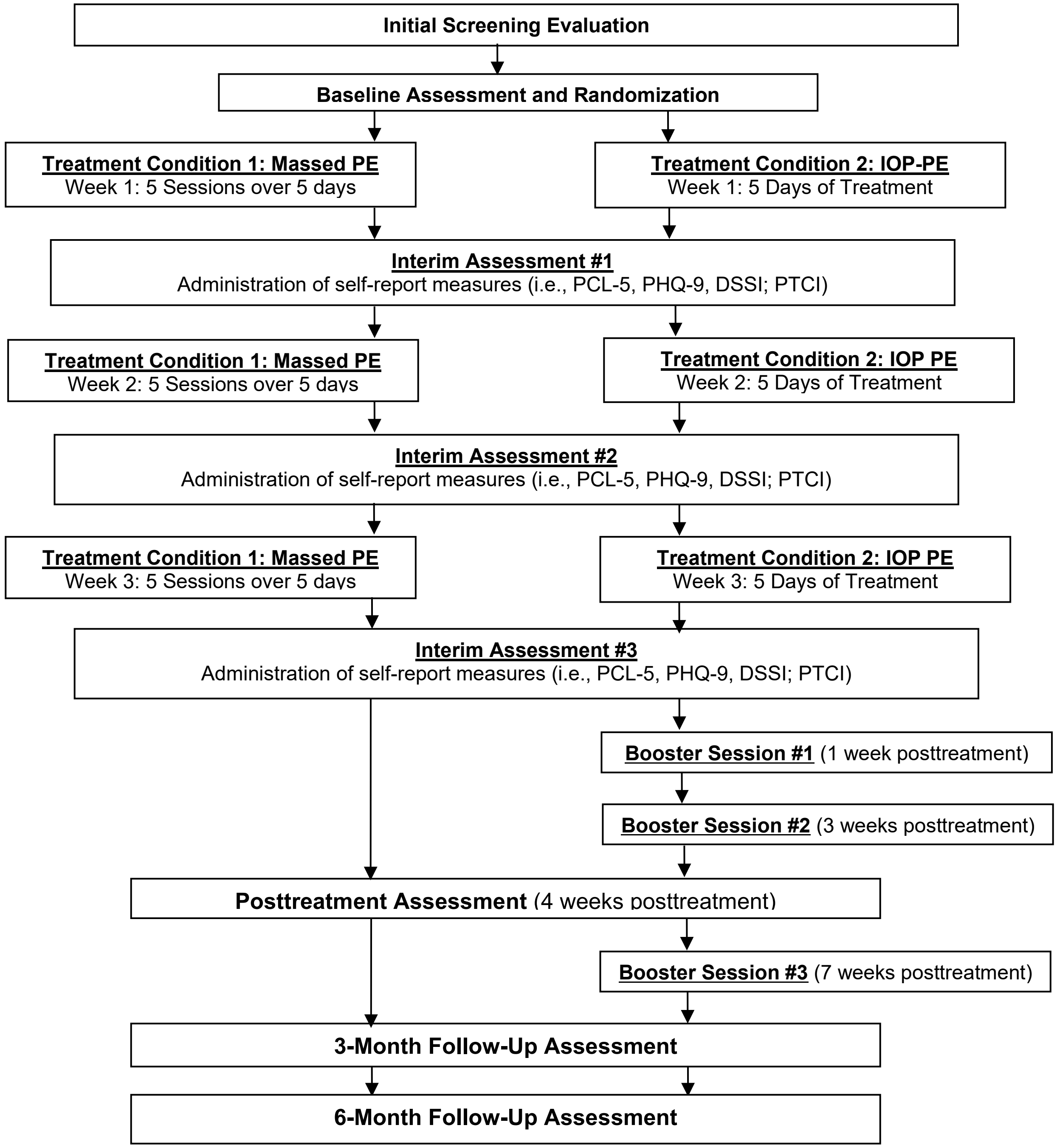

7.1.4. Description of the Recruitment and Prescreening Process.
Potential participants will be recruited at Joint Base San Antonio – Fort Sam Houston, San Antonio, TX; the South Texas Veterans Health Care System (STVHCS), San Antonio, TX; Fort Hood, TX or Waco, TX, and the surrounding communities. In general and at each of the performance sites, providers can obtain consent to contact from interested individuals for the study staff to follow-up. Providers can also give their patients contact information for the study staff so that interested individuals may contact study staff directly. Or, potential participants can self-refer in response to recruitment flyers distributed to various health care providers and posted in locations in the military and VA medical centers and at locations on military bases and in the community frequented by service members and veterans (see

Appendix B). Primary locations on bases include the Outpatient Behavioral Health Clinics, fitness centers, chapels, barracks, commissaries, and the PX. Potential participants may also self-refer in response to recruitment information on the STRONG STAR website. Interested persons can call or walk in to the STRONG STAR offices. STRONG STAR also recruits using various social media sites (e.g. Facebook, Twitter, LinkedIn, etc.) and web search engines (e.g. Google Ads, Bing, etc.). In addition, there may be events where information about STRONG STAR studies is provided and those interested may fill out a “consent to contact” form indicating that they would like a member of the research team to contact them at a later date to learn more about the study and schedule or complete pre-screening. Research staff will field incoming phone calls and walk-ins.

At SAMMC where the On-Site PI is engaged in research interacting with potential participants and participants, the On-Site PI will assist in recruitment and pre-screening in the ways described above. The On-Site PI is the Chief of the SAMMC Intensive Outpatient Program for Posttraumatic Stress Disorder (IOP for PTSD). The Chief and his staff will refer interested individuals from the SAMMC IOP for PTSD directly to the project team embedded in the SAMMC IOP Clinic using the hospital’s electronic referral system. The SAMMC IOP Chief and On-Site PI will maintain visibility of participant behavioral health services during the project timeline and will co-sign study therapist notes in the participant’s AHLTA electronic medical record.

At CRDAMC where there will be an On-Site Collaborator rather than On-Site PI who is not engaged in research activities. Service members at CRDAMC will be recruited through the recruitment procedures identified above and will be consented, assessed, and treated at the STRONG STAR Offices located on Fort Hood.

At the South Texas Veterans Health Care System (STVHCS), providers will refer veterans to the study or obtain consent to contact from interested individuals so that study staff can contact them directly about study participation. Potential participants that have been recruited from the STVHCS will be seen at the UTHSCSA Northwest Center (NWC) STRONG STAR – CAP offices to provide informed consent and complete study procedures.

Research activities to be conducted at the Central Texas Veterans Health Care System, Doris Miller VA Medical Center, VISN 17 Center of Excellence for Research on Returning War Veterans will be reviewed and monitored by the CTVHCS IRB.

Under an IRB approved HIPAA Waiver of Authorization, Alteration of Informed Consent, and Waiver of Documentation of Informed Consent, study personnel will conduct a brief telephone pre-screening where the basic study inclusion and exclusion criteria will be reviewed to help the individual determine if he or she meets the study criteria or has obvious exclusions from the study protocol so as to prevent individuals from making unnecessary travel for consent and more in-depth screening (see Appendix C). This information will be entered into a secure database as a phone call to a potential participant or a phone call from a potential participant: name, phone number, name of study the caller is interested in, referral date, referral source, potential eligibility status, reason if not eligible, and verbal permission to contact the caller in the future for other studies. We will also record the date and time of the call, outcome of the call, and any notes. Subjects who agree to study participation will sign a consent document before any further screening will take place. Any individually identifiable information and Protected Health Information (PHI) collected on individuals who do not consent to participation will not become part of the research data. If participants agree to participate in the research, the identifiable data collected will become part of the participants’ research records and will be stored according to the research confidentiality plan.

Service members or veterans who phone screen out from other IRB-approved STRONG STAR protocols will be offered the opportunity to be phone screened for participation in this study. If interested, a member of the research team will review eligibility with these potential participants (e.g., pre-screen) over the phone. If the person believes they may qualify for the study, the participant will be scheduled for an appointment in which consent will be obtained, and if authorized, the first baseline assessment will be completed.

7.1.5. Consent Process.

During the consent appointment, potential participants will have the study explained to them in a private location. The potential participant will be given a copy of the informed consent document (ICD) to read. After the potential participant has read the ICD, they will be given the opportunity to take the consent home to discuss the research with family and friends. The Research Team will be available to answer any questions about the research. Once the potential participant has reached a decision, the advising staff member will review the risks and benefits of the study and ensure the participant understands the research. The advising staff member will have the participant sign the consent form. A copy of the signed ICD will be given to the participant. As described in the consent, participants on active duty will have the option of having their Command notified by the Research Staff to ensure active duty Service Members are afforded the time to participate in the study. Command

agreement to allow for duty time to participate in this study is not a requirement for study participation. The advising staff member will document the informed consent process in the medical record of the participant. Baseline assessment will occur after consent.

Individuals who will need to travel a significant distance and reside temporarily in the local area where treatment is being provided from the study, will be provided the options of completing the consent appointment through audio teleconferencing. With their permission, an electronic copy of the consent form will be emailed to them for their review. As with in-person consent appointment, these individuals will be provided an opportunity to discuss the research with family and friends. Should they choose to participate, they will be asked to sign the consent form and email or fax it back to the study team. A baseline assessment will then be scheduled after consent. Individuals who are traveling significant distances to participate in treatment then will be provided the option to complete the initial screening baseline electronically and through audio teleconferencing in order to decrease the burden associated with traveling for those who do not meet inclusion/exclusion criteria.

7.1.6. Subject Screening Procedures.

Once the consent is signed, participants will then be asked to fill out the packet of assessments (see measures section below) with the Independent Evaluator (IE). The initial consent and screening will require up to 4 hours. This will include the completion of the questionnaires and interviews outlined in the table in Section 7.3 below. If the participant has been referred from another STRONG STAR study and already undergone baseline testing within the past 30 days, the participant will be asked as part of the consent process to use these assessments rather than repeating the assessment battery. If the participant is newly referred to this study, if it has been more than 30 days since baseline testing for another study, or the participant declines use of previously completed assessments, he or she will meet with an evaluator and complete the full baseline assessment per protocol.

The study team, with permission from the participant, will work with the Service Members' commander to secure a medical release of duty. The Commander will also be notified of the treatment and assessment schedule after randomization. As appropriate, the study team will also work with Veteran's civilian employers to maximize their ability to engage in treatment. For Service Members or veterans not meeting study inclusion criteria, the Study Staff will coordinate appropriate follow-up outside of the study.

7.1.7. Compensation for participation:

Participants will not be compensated for participation in assessment or treatment visits. All participants will be paid $25 each time they get their blood drawn. Participants who choose to withdraw prior to study completion will receive compensation for blood draws that they have completed. Payment will be provided via a rechargeable MasterCard® ClinCard. The MasterCard® ClinCard is a debit card issued to the study participant. Funds are loaded onto card through the ClinCard website at www.clincard.com. Only authorized users will be able to access the ClinCard website to add funds with a username and password. The ClinCard funds will be available to recipients within 1 business day and can be used as the participant chooses. The participant will be notified that their name, address and date of birth will be shared with a third-party (ClinCard) solely for the purposes of payment processing. This information will only be used for the administration of the payment and will be kept strictly confidential.

For veterans who are Texas residents and who live outside of the local area, reimbursement for mileage and meals will be provided. Car rental or airfare and lodging will also be provided at no cost to them. All other participants are required to pay for their own transportation to and from the clinic whenever they are scheduled to attend a visit.

7.1.8. <u>Treatment Procedures.</u>

PE for PTSD (Foa, Hembree, & Rothbaum, 2007) is an empirically supported behavioral therapy that utilizes exposure-based interventions to targets the psychological mechanisms (i.e., avoidance; maladaptive cognitive changes) thought to maintain trauma-related symptoms. A standard outpatient PE protocol (90 minute sessions, over 10 weeks) includes: a) providing a treatment rationale; b) providing psychoeducation on common reactions to trauma; c) teaching relaxed breathing; d) completing *in vivo* exposures; e.) conducting imaginal exposure; and f) facilitating emotional and cognitive processing for the trauma. With *in vivo* exposures, participants repeatedly and systematically approach realistically safe people, places, objects, and situations that they are currently avoiding. In imaginal exposures, participants repeatedly and systematically approach their trauma memory and related thoughts and feelings. PE serves as the foundation for both treatment conditions described. Ways in which PE for PTSD is being augmented for each condition in this study are outlined below. See Appendix A for the treatment manuals.

Massed-Prolonged Exposure (Massed-PE)

Massed-PE will include all of components of the Massed-PE protocol at Fort Hood with two enhancements:

1. Increased Number of Treatment Sessions. The proposed Massed-PE treatment will include a 50% increase in the number of treatment sessions of PE (15 sessions, 90 minutes each). The participants will have 3 consecutive weeks to complete treatment; however, the treatment window may be extended another week if necessary.

2. Release from Duty. The Massed-PE treatment will allow additional time to complete out-of-session PE homework exercises on a daily basis. The daily Massed-PE schedule during weekdays will begin with a 90-minute individual PE treatment session. The final portion of each daily treatment session will involve planning and scheduling of the homework exercises to be completed throughout the remainder of the day. Participants will complete these daily homework sessions as outlined in the primary treatment manual (Foa et al., 2007) without additional feedback or assistance from a therapist. The prescribed daily individual homework will include (1) listening to the 90-minute audiotape of that day's PE session, (2) completing at least two 45-minute in vivo exposure exercises, and (3) practicing relaxed breathing exercises. The in vivo exposure exercise may take 1-2 hours if it involves traveling to and from a specific location (e.g., shopping mall, movie theater, restaurant, sporting event, etc.) alone or with another individual (e.g., spouse, friend, therapist, etc.). Therefore, the overall amount of time devoted to PE and related homework exercises on a daily basis for the Massed-PE treatment on weekdays is estimated to be 6 to 7 hours. On the two weekends that occur during the middle of the 3-week Massed-PE, participants will be instructed to complete all of the regular homework exercises. The one exception for the weekends is that the participants will only listen to the 45-minute imaginal exposure portion of the treatment session audiotape from the previous Friday's individual PE treatment session rather than the full 90-minute treatment session. This is the standard PE protocol for review of session audiotapes between spaced-PE treatment sessions. In addition, weekend in vivo exercises may require traveling longer distances and require more time than may be feasible during weekdays.

Intensive Outpatient Program Prolonged Exposure (IOP-PE)

The IOP-PE will include the same primary treatment components as the Massed-PE protocol plus six augmentations to maximize outcomes and target military-relevant components not previously evaluated. Similar to the Mass-PE, participants will have 3 consecutive weeks to complete treatment; however, the treatment window may be extended another week if necessary.

1. Clinic-Based Completion of Daily Homework Assignments. Participants in the IOP-PE arm will complete all of their homework assignments in the IOP clinic, with the exception of in vivo exposure exercises. Patients will be provided with a private clinic office listen to the 90-minute PE session audiotape. Listening to audiotapes of treatments sessions, especially the imaginal exposure portion, is considered to be a key component of PE therapy. However, this component can be emotionally difficult and it is sometimes avoided. Providing structure and support to listen to the audiotape in the clinic will help ensure the completion of this PE component. Participants will be encouraged to take notes while listening to the audiotape about any portion that is impactful to them or that they would like to discuss during the brief processing session to be held with a therapist immediately after reviewing the audiotape (see below). Pilot data using this approach has demonstrated that this requires only about 15 minutes of additional therapist time with the potential to significantly improve outcomes.

2. Brief Therapist Feedback Sessions. Therapists will provide two 15-minute feedbacks sessions. The first will be after in vivo exposure homework to review any difficulties or challenges that may have occurred and to plan for the next in vivo exercise. The second will be after the listening to the PE session audiotape to focus on cognitive and emotional processing of the session.

3. Enhanced Social Support. Participants will be asked to identify a support person to be involved in treatment. Some participants may decline to identify a support person; in these instances the participant will be allowed to remain in the study although this element of treatment will not be administered. If the patient identifies a spouse or other support person, and if the support person is available and willing to attend, they will be invited to participate in Session 2 of the IOP-PE to review (1) the overall rationale for PE, (2) the schedule and format of the IOP-PE, (3) the Common Reactions to Trauma handout, and (4) methods to provide social support for the completion of in vivo exercises. This session will be completed by telephone or video if the support person is at a remote location. The support person will not be a study participant; no data will be collected from the support person only for the research.

4. Top Three Traumas. The IOP-PE arm will specifically assess and treat the top three traumas rather than limiting the imaginal exposure to the single worst and most currently distressing trauma.

5. Graduated Imaginal Exposure. The imaginal exposure portion of the IOP-PE arm will start with the trauma reported by the patient to be the least difficult of the top three traumas. It will then progress gradually to the worst trauma.

6. Posttreatment Booster Sessions. The IOP-PE treatment will include three booster sessions (up to 1-hour in length) scheduled for 1 week, 3 weeks, and 7 weeks after the completion of treatment. Booster sessions will be conducted in-person for participants in the local area and by telebehavioral health for those who traveled to participate in the research

study. The target of the booster sessions will be the maintenance and enhancement of treatment gains and the titration of in vivo exposure sessions into the patient's home environment.

While receiving treatment in the study through the 6-month follow-up assessment, participants will be asked not to work with another therapist or seek additional treatment for PTSD symptoms. Participants will also be asked not to change the dose or frequency of any medications they may have been prescribed for their posttraumatic stress symptoms as much as possible so that we can test the effect of the interventions rather than the effect of any change in medication.

7.2 Drugs, Dietary Supplements, Biologics, or Devices. N/A

7.3. Study Procedures/Research Interventions.

Study Procedures	Base Line	Post-BL	Wk 1: Session 1	Interim Assess #1	Interim Assess #2	Interim Assess #3	Post-TX	3-Mo FU	6-Mo FU
Informed Consent	X								
Randomization		X							
1. Clinician Administered PTSD Scale (CAPS-5)	X						X	X	X
2. PTSD CheckList-5 (PCL-5)	X			X	X	X	X	X	X
3. Sheehan Disability Scale	X						X	X	X
4. Brief Inventory of Psychosocial Functioning	X						X	X	X
5. Demographics & Military Service Characteristic	X								
6. Combat-Related PTSD Beliefs Inventory	X								
7. History of Head Injuries	X						X	X	X
8. Health Questionnaire	X						X	X	X
9. MINI -7.0 (Mini International Neuropsychiatric Interview for DSM-5) Psychosis and Mania modules only.	X								
10. Alcohol Use Disorders Identification Test (AUDIT)	X								
11. Deployment Risk and Resilience Inventory (DRRI-2) Subscales:	X								

- Combat Experience<br>- Post-battle Experiences									
12. Depressive Symptoms Index-Suicidality Subscale (DSI-SS)	X			X	X	X	X	X	X
13. Self-Injurious Thoughts and Behaviors Interview (SITBI)	X						X	X	X
14. Patient Health Questionnaire-9 (PHQ-9)	X			X	X	X	X	X	X
15. Generalized Anxiety Disorder Screener (GAD-7)	X						X	X	X
16. Veterans Rand 12-Item Health Survey (VR-12)	X						X	X	X
17. Dimension of Anger Reactions-5 (DAR-5)	X						X	X	X
18. Conflict Tactics Scale - Revised (CTS2)	X						X	X	X
19. Quick Drinking Screen (QDS)	X						X	X	X
20. Response to Stressful Experiences Scale (RSES)	X						X	X	X
21. Life Event Checklist (LEC - 5)	X						X	X	X
22. PTSD Diagnostic Scale for DSM-5 (PDS-5)	X						X	X	X
23. DRRI-2 Post-Deployment Social Support subscale	X						X	X	X
24. Patient Health Questionnaire (PHQ)-15	X						X	X	X
25. Trauma-Related Guilt Inventory (TRGI)	X						X	X	X
26. Post-Traumatic Cognitions Inventory (PTCI)	X			X	X	X	X	X	X
27. Childhood Trauma	X								

Questionnaire (CTQ)									
28. Insomnia Severity Index (ISI)	X						X	X	X
29. PROMIS Sexual Function and Satisfaction Measures	X						X	X	X
30. PROMIS Sleep Disturbance and Sleep-Related Impairment short forms	X						X	X	X
31. Snoring, Tired, Observed, Blood Pressure (STOP) Sleep Apnea Screen	X								
32. Fagerstrom Test for Nicotine Dependence (FTND)	X						X	X	X
33. Fagerstrom Test for Nicotine Dependence – Smokeless Tobacco (FTND-ST)	X						X	X	X
34. Credibility / Expectancy Questionnaire (CEQ)			Session 2				X	X	X
35. Independent Evaluator Blind Form							X	X	X
36. Blood for genetic biomarkers		X		X	X	X			

7.3.1 Collection of Human Biological Specimens:
Blood will be collected prior to treatment, and during three of the sessions of the 3-week treatment phase for all participants in collaboration with the CAP-Biomarkers and Genomics Core to examine gene expression profile links with Intensive Outpatient Prolonged Exposure that may be predictive of treatment outcomes. A skilled phlebotomist will draw the samples. All blood samples will be collected at the study site.

7.3.1.1 Laboratory evaluations and special precautions:
All blood samples will be collected at the study site and then transported to the CAP Biomarkers and Genomics Core in the UTHSCSA Department of Psychiatry and Behavioral Sciences at the UT Health-Long School of Medicine in room 727F for processing and storage in accordance with the CAP Biomarkers and Genomics SOP. This location is monitored 24-7 by a wireless monitoring system on the Isensix system and has approval as a VA-Approved Tissue Bank. The study staff will be available to offer assistance if the participant is distressed at any point during any procedures.

7.3.1.2 Specimen storage:
Biological specimens will be coded with a digital bar code that can be linked to the study and subject only by key-codes maintained by the CAP Data Management and Biostatistics Core.

7.3.2 Data Collection.

7.3.2.1 Instrumentation: See the table at Section 7.3 above for a summary of the assessments and timing of administration. A description of each of the assessments can be found at the end of this protocol. Assessments will be administered in person whenever possible. However, in order to accommodate participant schedules and/or instances in which a participant does not reside in the local area at the time of a follow up assessment, we may collect full or partial assessments in person or via phone, telebehavioral health, or electronic data capture using a secure link to the encrypted STRONG STAR database. Assessments completed remotely will not include biospecimen collection. Reasonable efforts will be made to collect all data as described in this protocol, but we expect some participants may not be able to complete part or all of any given follow up assessment.

The following data collected at Baseline, Post-Treatment, and 3-Month Follow-up will contribute to a separate study titled *Hypertension Risk in Deployed OIF/OEF/OND Veterans* being conducted at Yale University School of Medicine by Matthew M. Burg, PhD under the Yale IRB approval number 1506015992 and UTHSCSA IRB approval number HSC20170630H: History of Head Injuries, Clinician Administered PTSD Scale (CAPS-5), PTSD CheckList-5 (PCL-5), Alcohol Use Disorders Identification Test (AUDIT), and Veterans Rand 12-Item Health Survey (VR-12). This data will only be shared if the participant consents to participate in the separate study.

Clinician Administered PTSD Scale (CAPS-5) data collected at Baseline, Post-Treament, and 3-Month Follow-up will also contribute to a separate study titled *Neurobiological Predictors and Mechanisms In Exposure Therapy for PTSD* being conducted at Carl R. Darnall Army Medical Center (CRDAMC) and UTHSCSA under UTHSCSA IRB protocol number HSC20170714H. The Overall PI for HSC20170714H is Sheila Rauch, PhD at Emory University. This data will only be shared if the participant consents to participate in the separate study.

7.3.2.2 Data Storage and Access: Data will be coded using an assigned number. Hard copies of data collected during treatment will be placed into a lock box which will be transported by car to University of Texas Health Sciences Center San Antonio (UTHSCSA) STRONG STAR offices by a STRONG STAR staff member who will place it into the locked cabinets at the STRONG STAR offices. Data will be entered into the STRONG STAR database on a secure UTHSCSA server (physically located at the UTHSCSA Advanced Data Center), by member of the research team. Electronic data will be stored, managed, and analyzed by the STRONG STAR – CAP Data and Statistics Core staff of the STRONG STAR consortium. The study activities accomplished by the CTVHCS employees will be reviewed and monitored by the CTVHCS IRB.

Every member of the Research Team will be trained and monitored about how to handle and protect both medical and research records. Furthermore, the Research Team strictly controls access to study data.

7.4 Statistical Consideration

7.4.1 Sample Size Estimation. The primary hypotheses are tests of treatment differences in pre-post means using the pre-treatment score as a covariate. Statistical power for the analysis of covariance with r = .50 between baseline and post-treatment is .81 to detect a standardized mean difference of .35. An analysis of unadjusted pre-post difference scores with *N* = 100 per group would have power = .80 for a standardized difference of about d = .40. Cohen (1988) defined d = .2 as "small," and d = .5 as "medium." The PCL-5 in our experience has a baseline standard deviation of about 15, and smaller differences than d = .35 are unlikely to be clinically meaningful. For differences in diagnostic proportions (Hypothesis 2), *N* = 100 gives power = .80 for a difference of about 20% (i.e., 45% vs. 65% or 50% vs. 70%). This is an odds ratio of about 2.3, roughly equivalent to Cohen's *d* = .45. For bivariate correlations, *N* = 100 has power = .88 to detect a medium r = .30.

7.4.2 Primary (i.e., primary outcome variables) and secondary endpoints.

The study aim is to evaluate the efficacy of IOP-PE relative to Massed-PE in the reduction of PTSD diagnoses and symptom severity (primary measures = CAPS severity score; PCL-5) and associated disability and psychosocial functioning (Sheehan Disability Scale; Brief Inventory of Psychosocial Functioning). Secondary endpoints include associated psychopathology (severity scores on measures of depression, general anxiety, anger, and PTSD-related cognitions).

7.4.3 Data analysis.

7.4.3.1 General Statistical Considerations.

Data will be entered into the STRONG STAR database on a secure UTHSCSA server meeting HIPAA requirements on an ongoing basis using specialized software that provides for double entry and numerous internal validity checks and produces regular reports on data flow to identify any ongoing recruitment issues. Prior to statistical analyses, data will be inspected for outliers, unusual distributions that suggest transformations such as log, and patterns of missing data. Data will be analyzed using the SAS statistical system (SAS 9.4).

Missing Data. An Intent-to-treat analysis will be used with a likelihood-based analysis method which is robust if data are missing at random. Outcome data will be collected on all participants randomized, even if they elect to end treatment or study participation. If a reasonable predictive model for missing data can be found and patterns of attrition suggest possible bias, the primary analyses will be supplemented with inverse propensity score weighting and multiple imputation (Hirano et al., 2003, Lunceford & Davidian, 2004). This approach will make it possible to perform sensitivity analyses (Allison, 2014; Dziura et al., 2013; Mallinckrodt et al., 2012). All analyses will be implemented in the latest release of SAS/STAT PROC MI in SAS version 9.4 with the inclusion of a new Not Missing at Random option (Demirtas & Schafer, 2003; Little, 2009; Enders, 2010).

Baseline Analyses and Sites. Randomization is expected to produce a high degree of similarity between treatment groups, but treatment groups will be compared at baseline to determine comparability on the outcome variables and to identify potentially important covariates that are not of primary interest in the hypotheses but relate to the outcome criteria. Baseline scores will be used as covariates. Differences between active duty or veteran status and interactions of status by treatment are not expected once relevant patient characteristics such as baseline severity are controlled, but preliminary analyses will consider the need to include site in the analysis designs.

7.4.3.2 Hypotheses and Specific Data Analysis Plans

Hypothesis 1: The IOP-PE treatment will result in larger reductions than the Massed-PE treatment in clinician-assessed PTSD symptoms on the CAPS-5 and self-reported PTSD symptoms on the PCL-5 at the posttreatment, 3-month, and 6-month follow-up points.

Hypothesis 2: The IOP-PE treatment will result in a larger percentage of participants who no longer meet clinician-assessed PTSD on the CAPS-5 than the Massed-PE treatment at the posttreatment, 3-month, and 6-month follow-up points.

Hypothesis 3: The IOP-PE treatment will result in greater improvements than the Massed-PE treatment in functional outcomes on the Sheehan Disability Scale and the Brief Inventory of Psychosocial Functioning at the posttreatment, 3-month, and 6-month follow-up points.

The analysis method for Hypotheses 1-3 will be mixed effects regression models with repeated measures. The general linear mixed model is appropriate for dimensional measures that are approximately normally distributed such as the CAPS-5 and PCL-5 (Hypothesis 1) and measures of self-reported symptoms (Hypotheses 3). For diagnosis (Hypothesis 2), the generalized linear mixed model is appropriate specifying binomial error and a logit link function. Fixed effects are treatment (Massed-PE vs. IOP-PE), time (post-treatment, 3 & 6 month FU), and the interaction of treatment by time, with baseline level used as a covariate when available. Planned contrasts will be done to test the significance of pre-post change within each treatment. The primary tests for Hypotheses 1 and 3 will be the difference between treatments in these pre-post change scores. For Hypothesis 2, it is the difference in proportion remitted post-treatment (all participants have PTSD pre-treatment). Stability of outcomes will be examined by evaluating the significance of changes after treatment and comparing baseline to the follow-up scores in each treatment condition. It is hypothesized that similar improvements will be found in service members and veterans, but the study is not powered to detect significant noninferiority differences.

7.4.3.3 Statistical Power. The primary hypotheses are tests of treatment differences in pre-post means using the pre-treatment score as a covariate. Statistical power for the analysis of covariance with $r = .50$ between baseline and post-treatment is .81 to detect a standardized mean difference of .35. An analysis of unadjusted pre-post difference scores with $N = 100$ per group would have power = .80 for a standardized difference of about $d = .40$. Cohen (1988) defined $d = .2$ as "small," and $d = .5$ as "medium." The PCL-5 in our experience has a baseline standard deviation of about 15, and smaller differences than $d = .35$ are unlikely to be clinically meaningful. For differences in diagnostic proportions (Hypothesis 2), $N = 100$ gives power = .80 for a difference of about 20% (i.e., 45% vs. 65% or 50% vs. 70%). This is an odds ratio of about 2.3, roughly equivalent to Cohen's $d = .45$. For bivariate correlations, $N = 100$ has power = .88 to detect a medium $r = .30$.

7.7 Confidentiality.

All in-person therapy sessions and interview assessments will be delivered in private offices at either the San Antonio Military Medical Center, the STRONG STAR Clinic at Ft Hood, the STRONG STAR Clinic at the UTHSCSA, or the Doris Miller VA Medical Center, VISN 17 Center of Excellence for Research on Returning War Veterans, Waco, Texas. Data will be stored by an assigned participant code number so that data records and specimens can be viewed by password-authenticated, authorized investigators and Consortium personnel. Digital audio recordings of assessments or video recordings of PE sessions will be labeled with the participant's study id number and saved on a secure password protected server. Those recordings to be reviewed for fidelity to ensure that the treatment is being delivered in accordance with the treatment manual will be viewed on a secure password protected server. There is no option for the reviewers to download or otherwise save the recordings to their computers. Every member of the Research Team will be trained and monitored about how to handle and protect both medical and research records. Only authorized study staff, and members of the STRONG STAR Biostatistics and Data Management Core staff will have access to either the raw data or electronic study data.

7.7.1 Certificate of Confidentiality.

We are not seeking a Certificate of Confidentiality.

7.7.2. Data Protection.

Data will be coded using an assigned number. Data collected during treatment will be placed into a locked filing cabinet and stored securely in a locked room by a STRONG STAR staff member. Files will be kept securely at the University of Texas Health Science Center (UTHSCSA) STRONG STAR offices in either San Antonio or Fort Hood depending on where the research activities occur. Research files from Waco Texas will be transported in a locked box by car to the Fort Hood location by a STRONG STAR staff member, where they will be stored securely. Audio and videotapes will be uploaded to the secure STRONG STAR data base. The STRONG STAR data server is physically located at the Advanced Data Center (ADC) has 24x7 onsite security, card key, biometric access controls and video surveillance. University of Texas Health Science Center at San Antonio (UTHSCSA) ADC facility also maintains Gen 2 firewall devices to protect and prohibit any unauthorized access to UTHSCSA data. All UTHSCSA network devices are monitored by state of the art monitoring applications that include configuration audit, management, and availability 24x7. Every member of the Research Team will be trained and monitored about how to handle and protect both medical and research records. Furthermore, the Research Team strictly controls access to study data. Local study sites will maintain a list of assignment numbers for the purpose of linking subsequent research materials.

A Data Safety and Monitoring Plan (DSMP) has been developed in accordance with the National Institutes of Health Office of Human Research Protection to assure the appropriate clinical safety monitoring of study subjects participating in this study.

7.7.3. Long Term Data Storage.

A *STRONG STAR Repository* has been approved by both the UTHSCSA (HSC20100475H) and BAMC (C.2011.054d; IRBNet #363444) IRBs to enable the STRONG STAR Consortium to store specimens and data for future use. The STRONG STAR Repository will create a large comprehensive database of information, biological specimens and neuroimages related to the identification, assessment, and treatment of PTSD in our active duty and retired veterans of conflicts following 9-11. All information entered into the STRONG STAR Repository will be extracted from primary datasets collected as part of IRB-approved studies, including this study, being conducted and /or supported by the projects of the STRONG STAR Consortium. These study databases will be established and maintained by the Biostatistics and Data Management Core of the STRONG STAR Consortium. A unique, sequential alpha-numeric STRONG STAR ID will be assigned to each participant at the time of recruitment into this study. However, all Repository data will be identified with a different code number that can be cross linked to the original study code only through records maintained by the STRONG STAR Biostatistics and Data Management Core. Data, biological specimens and images will constitute the STRONG STAR PTSD Repository. Participation in the repository will be completely voluntary and entirely optional which means that a potential participant's willingness to participate in the repository has no influence upon their eligibility to participate in the primary STRONG STAR study they have either already enrolled in or are considering enrolling in. At the conclusion of this study, participants who signed the consent to have their specimens and data placed in the STRONG STAR Repository will be maintained under the IRB-approved Repository protocol. Biological specimens and information from study participants who declined participation in the STRONG STAR Repository will be permanently de-identified (i. e., all PHI will be deleted from the study data bases) and the de-identified blood and information placed in the STRONG STAR Repository for future use.

8.0 <u>RISKS/BENEFITS ASSESSMENT</u>

8.1 Risks.

Likely, but Not Serious Risks

- Becoming emotionally upset or experiencing an initial increase of PTSD symptoms due to the consideration of traumatic events. Studies with PE suggest that a small minority experience an increase in symptoms of PTSD after the initiation of imaginal exposure exercises and for this minority, the distress and increased symptoms are temporary, are not predictive of poor outcome, and are not associated with increased likelihood of dropout (Foa et al., 2002).
- Discomfort, bruising or both, at the site of the needle puncture during blood sample collection.
- Some people experience fainting, the formation of a small blood clot or swelling of the vein and surrounding tissue, or bleeding from the puncture site.
- This research includes genetic research with the participant's samples. Using new technology, information about DNA structure (genetic information) can be used to indicate risk for developing certain diseases. This genetic information is unique to the participant and may indicate changes in their future health status or life expectancy, or that of their children and other relatives. If released, these discoveries could be stressful and cause psychological difficulties or family problems.

Rare, but Serious Risks

With the handling of medical and research records there is always the possibility of a breach of confidentiality. However, every effort is made to protect the privacy of participants. Every member of the Research Team is carefully trained and monitored about how to store, handle, and protect participant records.

Risks of PTSD Diagnosis regardless of Treatment

Possibility of increased suicidal risk. One of the risks of PTSD both in and out of treatment is attempted suicide, which can result in death.

Safeguards for Protecting Participants

For urgent issues, participants will be instructed to get help immediately by going to the Emergency Department open 24 hours at, University Hospital, SAMMC, CRDAMC, Doris Miller VA Medical Center, VISN 17 Center of Excellence for Research on Returning War Veterans, or STVHSC, depending on which site the participant is receiving care. Alternatively, participants will be advised to call the suicide hotline or may be connected directly to the hotline via third party connection, or go to a nearby civilian emergency room. Each week, the therapist will review the participant's progress and symptom levels and will provide individualized feedback to the participant as needed. Participants complete weekly self-report assessment measures that will assess PTSD severity, depressive symptoms, and suicidal ideation. This will allow therapists to closely monitor any increase in distress. Individualized safety planning will be utilized as needed by members of the treatment team.

Any indication that the participant is considering suicide will be handled following the STRONG STAR Consortium SOPs in concert with the local site-specific policies and procedures. Participants can be seen at the respective Military Treatment Facility (MTF) or VA Emergency Department at any time.

Research Monitor: The United States Army Medical Research and Material Command (USAMRMC) Human Research Protection Office (HRPO) determined this study to be greater than minimal risk and in accordance with DoDI 3216.02, a study Research Monitor has been appointed by the IRB who will oversee the safety of the research and report observations/findings to the IRB or a designated institutional official. The Research Monitor will review all unanticipated problems involving risks to subjects or others associated with the protocol and provide an independent report of the event to the IRB. The Research Monitor may discuss the research protocol with the investigators; shall have authority to stop a research protocol in progress, remove individual human subjects from a research protocol, and take whatever steps are necessary to protect the safety and well-being of human subjects until the IRB can assess the monitor's report; and shall have the responsibility to promptly report their observations and findings to the IRB or other designated official and the HRPO.

8.2 Potential Benefits.

Potential benefits of participation in this study may include a reduction in PTSD symptoms over the course of therapy. In addition, the knowledge gained from this study will serve to inform the most effective early interventions for the prevention and treatment of combat-related PTSD in active-duty military personnel and veterans.

8.3 Risk/Benefit Assessment: Potential benefits to participants are direct (expected reduction in PTSD severity along with reduction of general anxiety, depression, anger, and guilt) and indirect (enhancing our knowledge about the comparative efficiency and efficacy of different ways of delivering PE, as well as knowledge of underlying biological mechanisms of

treatment efficacy). The possible risks (i.e., temporary increase in distress and severity) associated with participation are reasonable in this context given the level of participant monitoring and access to research and clinical staff. We believe that the possible benefits from participating in this study outweigh the possible risks.

8.4 Alternatives: Mental health treatment is available at associated MTF and VA sites and includes various forms of psychotherapy and drug treatments. Service members or veterans can request treatment for PTSD through Army One-Source and may be eligible for care at one of the Veterans Healthcare System facilities or clinics. Not participating in the study is also an alternative.

9.0 ADVERSE EVENTS, UNANTICIPATED PROBLEMS, AND DEVIATIONS

9.1 Adverse Events will be assessed and monitored according to the established STRONG STAR and CAP SOP and the IRB of record's policies and procedures.

9.2 Reporting Adverse Events, Unanticipated Problems Involving Risks to Subjects or Others (UPIRSOs), and Deviations to the Office of the IRB.

All adverse events, unanticipated problems involving risk to subjects or others, and deviations will be reported to the Institutional Review Board (IRB) in accordance with current IRB policy. UPIRSOs and recurrent non-compliance with study procedures will be reported promptly to the IRB. Further, the study Research Monitor will review all unanticipated problems involving risks to subjects or others associated with the protocol and provide an independent report of the event to the IRB. All adverse events that do not meet the UPIRSO criteria and deviations that are not non-compliance will be summarized at Continuing Review per the IRB of record's policy.

10.0 WITHDRAWAL FROM STUDY PARTICIPATION.

Participation in the study may be discontinued by the principal investigator if continued participation is considered a danger to a participant's welfare. Reasons for discontinuation include: 1) a serious adverse event such that continued participation would be a danger to the participant; 2) clinical worsening for any reason that is deemed to necessitate non-study psychological or psychiatric treatment; 3) exacerbation of PTSD, anxiety, or depressive symptoms that the participant cannot tolerate; or 4) discontinuation would be in the participant's best interest. Participants deemed candidates for discontinuation will be discussed in the weekly conference calls with the therapist and the supervisor and will be brought to the attention of the PI for final decision.

Participants who are discontinued from the study for any reason will be scheduled for a final evaluation within one week and given appropriate treatment referrals. If participants are discontinued due to a serious adverse event, they will continue to be followed clinically by the therapist and/or member of the research staff until the adverse event is resolved or becomes stable. If participants are discontinued for a medical or psychiatric reason, they will be given the opportunity to either complete the balance of their IOP-PE or Massed-PE sessions or to receive a full course of IOP-PE or Massed-PE after the condition has resolved or stabilized and the endpoint assessment has been completed. The reason the participants are discontinued from the study and any referrals made will be documented. Participants will be told they will be contacted for follow-up whether or not they complete the trial.

11.0 REFERENCES.

Allison, P. (2014). Sensitivity analysis for not missing at random. Retrieved from http://statisticalhorizons.com/sensitivity-analysis

Bisson, J. I., Ehlers, A., Matthews, R., Pilling, S., Richards, D., & Turner, S. (2007). Psychological treatments for chronic post-traumatic stress disorder: Systematic review and meta-analysis. *British Journal of Psychiatry, 190,* 97-104. http://dx.doi.org/10.1192/bjp.bp.106.021402

Blount, T., Cigrang, J., Foa, E., Ford, H., & Peterson, A. (2013). Intensive outpatient prolonged exposure for combat-related PTSD: A case study. *Cognitive and Behavioral Practice, 21*, 89-96. http://dx.doi.org/10.1016/j.cbpra.2013.05.004.

Cigrang, J. A., Peterson, A. L., & Schobitz, R. P. (2005). Three American troops in Iraq: Evaluation of a brief exposure therapy treatment for the secondary prevention of combat-related PTSD. *Pragmatic Case Studies in Psychotherapy, 1*(2). http://dx.doi.org/10.14713/pcsp.v1i2.857

Cigrang, J. A., Rauch, S. A. M., Avila, L. L., Bryan, C. J., Goodie, J. L., Hryshko-Mullen, A., Peterson, A. L., & the STRONG STAR Consortium. (2011). Treatment of active-duty military with PTSD in primary care: Early findings. *Psychological Services 8*(2), 104-113. http://dx.doi.org/10.1037/a0022740

Cigrang, J. A., Rauch, S. A. M., Mintz, J., Brundige, A., Avila, L. L., Bryan, C. J., Goodie, J. L., Peterson, A. L., & the STRONG STAR Consortium (2015). Treatment of active duty military with PTSD in primary care: A follow-up report. *Journal of Anxiety Disorders, 36*, 110-114. http://dx.doi.org/10.1016/j.janxdis.2015.10.003

Cohen, J. (1988). *Statistical power analysis for the behavioral sciences (2nd ed.). New Jersey: Lawrence Erlbaum Associates.*

Department of Veterans Affairs. (2015a). *Analysis of VA health care utilization among Operation Enduring Freedom (OEF), Operation Iraqi Freedom (OIF), and Operation New Dawn (OND) veterans: Cumulative from 1st Qtr FY 2002 through 2nd Qtr FY 2015 (October 1, 2001 – March 31, 2015).*Washington, DC: Author. Retrieved from http://www.publichealth.va.gov/docs/epidemiology/healthcare-utilization-report-fy2015-qtr2.pdf

Department of Veterans Affairs. (2015b). *Report on VA facility specific Operation Enduring Freedom (OEF), Operation Iraqi Freedom (OIF), and Operation New Dawn (OND) veterans coded with potential or provisional PTSD: Cumulative from 1st Qtr FY 2002 through 2nd Qtr FY 2015 (October 1, 2001 – March 31, 2015).*Washington, DC: Author. Retrieved from http://www.publichealth.va.gov/docs/epidemiology/ptsd-report-fy2015-qtr2.pdf

Demirtas, H., & Schafer, J. L. (2003). On the performance of random-coefficient pattern-mixture models for non-ignorable drop-out. *Statistics in Medicine*, *22*, 2553–2575. http://dx.doi.org/10.1002/sim.1475

Dziura , J. D., Posta, L. A., Zhao, Q., Zhixuan, F., & Peduzzi P. (2013). Strategies for dealing with missing data in clinical trials: From design to analysis. *Yale Journal of Biology and Medicine*, *86*, 343-358. Retrieved from http://medicine.yale.edu/yjbm/

Enders , C. K. (2010). Models for not missing at random data. *Applied missing data analysis* (pp. 386-328). Guilford Press, New York, NY.

Foa, E., Cashman, L., Jaycox, L., & Perry, K. (1997). The validation of a self-report measure of PTSD: The Posttraumatic Diagnostic Scale. *Psychological Assessment, 9*, 445-451.

Foa, E. B., Hembree, E. A., & Rothbaum, B. O. (2007). *Prolonged exposure therapy for PTSD: Emotional processing of traumatic experiences: Therapist guide.* New York, NY: Oxford University Press.

Foa, E. B., Yadin, E., McLean, C. P., Young-McCaughan, S., Mintz, J., Litz, B., Dondanville, K., Yarvis, J. S., & Peterson, A. L. (2015, August). *Efficacy of a 2-week massed Prolonged Exposure protocol for PTSD among active duty military personnel.* Poster presented at the Military Health System Research Symposium, Fort Lauderdale, FL.

Foa, E. B., Zoellner, L. A., Feeny, N. C., Hembree, E. A., & Alvarez-Conrad, J. (2002). Does imaginal exposure exacerbate PTSD symptoms?. Journal Of Consulting And Clinical Psychology, 70(4), 1022-1028. doi:10.1037/0022-006X.70.4.1022

Hirano, K., Imbens, G.W., & Ridder, G. (2003). Efficient estimation of average treatment effects using the estimated propensity score. *Econometrica, 71*, 1161-1189. http://dx.doi.org/10.1111/1468-0262.00442

Hoge, C.W., Auchterlonie, J. L., & Milliken, C. S. (2006). Mental health problems, use of mental health services, and attrition from military service after returning from deployment to Iraq or Afghanistan. *Journal of the American Medical Association, 295*, 1023-1032. http://dx.doi.org/10.1001/jama.295.9.1023

Institute of Medicine. (2014). *Treatment for posttraumatic stress disorder in military and veteran populations: Final assessment.* Washington, DC: The National Academies Press.

Little, R. (2009). Selection and pattern-mixture models. In G. Fitzmaurice, M. Davidian, G. Verbeke, & G. Molenberghs (Eds.). *Longitudinal data analysis* (pp. 409-432). Boca Raton, FL: Chapman & Hall/CRC Press.

Lunceford, J. K., & Davidian, M. (2004). Stratification and weighting via the propensity score in estimation of causal treatment effects: A comparative study. *Statistics in Medicine, 23*, 2937-2960. http://dx.doi.org/10.1002/sim.1903

Mallinckrodt, C. H., Lin, Q., & Molenberghs, M. (2012). A structured framework for assessing sensitivity to missing data assumptions in longitudinal clinical trials. *Pharmaceutical Statistics*, *12*, 1-6. http://dx.doi.org/10.1002/pst.1547

Orsillo, S. M. (2001). Measures for acute stress disorder and posttraumatic stress disorder. In M.M. Antony & S.M. Orsillo (Eds.), *Practitioner's guide to empirically based measures of anxiety* (pp. 255-307). New York: KluwerAcademic/Plenum.

Peterson, A. L., Foa, E. B., & Riggs, D. S. (2011). Prolonged exposure therapy for combat-related PTSD. In B. A. Moore & W. Penk (Eds.), *Treating PTSD in military personnel: A clinical handbook* (pp. 42-58). New York, NY: Guilford.

Peterson, A. L., Luethcke, C. A., Borah, E. V., Borah, A. M., & Young-McCaughan, S. (2011). Assessment and treatment of combat-related PTSD in returning war veterans. *Journal of Clinical Psychology in Medical Settings, 18,* 164-175. http://dx.doi.org/10.1007/s10880-011-9238-3

Powers, M., Halpern, J., Ferenschak, M., Gillihan, S., & Foa, E. (2010). A meta-analytic review of prolonged exposure for posttraumatic stress disorder. *Clinical Psychology Review, 30,* 635-641. http://dx.doi.org/10.1016/j.cpr.2010.04.007

Resick, P. A., Williams, L. F., Suvak, M. K., Monson, C. M., & Gradus, J. L. (2012). Long-term outcomes of cognitive-behavioral treatments for posttraumatic stress disorder among female rape survivors. *Journal of Consulting and Clinical Psychology, 80*, 201-210. http://dx.doi.org/10.1037/a0026602

Richardson, L. K., Frueh, B. C., & Acierno, R. (2010) Prevalence estimates of combat-related post-traumatic stress disorder. *Australian & New Zealand Journal of Psychiatry, 44* (1), 4-19. http://dx.doi.org/10.3109/00048670903393597

Steenkamp, M. M., Litz, B. T., Hoge, C. W., Marmar, C. R. (2015). Psychotherapy for military-related PTSD: A review of randomized clinical trials. *Journal of the American Medical Association, 314,* 489-500. http://dx.doi.org/10.1001/jama.2015.8370
Sundin, J., Herrell, R. K., Hoge, C. W., Fear, N. T., Adler, A. B., Greenberg, N., Riviere, L. A., Thomas, J. L., Wessely, S., & Bliese, P. D. (2014). Mental health outcomes in US and UK military personnel returning from Iraq. *British Journal of Psychiatry*, *204*, 200-207. http://dx.doi.org/10.1192/bjp.bp.113.129569
Tan, M. (2015, July 10). Army lays out plan to cut 40,000 soldiers. *Military Times*. Retrieved from http://www.armytimes.com/story/military/pentagon/2015/07/09/army-outlines-40000-cuts/29923339/
Weathers, F. W., Blake, D. D., Schnurr, P. P., Kaloupek, D. G., Marx, B. P., & Keane, T. M. (2013). The Clinician-Administered PTSD Scale for DSM-5 (CAPS-5). Interview available from the National Center for PTSD at www.ptsd.va.gov.

12.1 Measurement Bibliography:

Adler, A. B., Litz, B. T., Castro, C. A., Suvak, M., Thomas, J. L. Burrell, L., McGurk, D., Wright, K.W., & Bliese, P.B. (2008). A group randomized trial of critical incident stress debriefing provided to U.S. peacekeepers. *Journal of Traumatic Stress, 21*, 253-263.
Babor, T. F., Higgins-Biddle, J. C., Saunders, J. B. & Monteiro, M. G. (2001). *The Alcohol Use Disorders Identification Test: Guidelines for Use in Primary Care, 2nd Edition.* Geneva, Switzerland: World Health Organization.
Bastien, C. H., Vallieres, A., Morin, C. M., (2001). Validation of the Insomnia Severity Index as an outcome measure for insomnia research. *Sleep Medicine, 2*(4), 297-307.
Batterham, P. J., Ftanou, M., Pirkis, J., Mackinnon, A. J., Beautrais, A., Fairweather-Schmidt, A. K., & Christensen, H. (2014, December 15). A systematic review and evaluation of measures for suicidal ideation and behaviors in population-based research. *Psychological Assessment.* Advance online publication. http://dx.doi.org/10.1037/pas0000053
Borkovec, T. D & Nau, S. D. (1972). Credibility of analogue therapy rationales. *Journal of Behaviour Therapy and Experimental Psychiatry, 3*, 257-260.
Buysse, D. J., Yu, L., Moul, D. E., Germain, A., Stover, A., Dodds, N. E., Johnston, K. L., Shablesky-Cade, M. A., & Pilkonis, P. A. (2010). Development and validation of patient-reported outcome measures for sleep disturbance and sleep-related impairments. *Sleep, 33,* 781-792.
Cameron, I. M., Crawford, J. R., Lawton, K., & Reid, I. C. (2008). Psychometric comparison of PHQ-9 and HADS for measuring depression severity in primary care. *British Journal of General Practice, 58,* 32-36.
Cella, D., Riley, W., Stone, A., Rothrock, N., Reeve, B., Yount, S., ... & Group, P. C. (2010). The Patient-Reported Outcomes Measurement Information System (PROMIS) developed and tested its first wave of adult self-reported health outcome item banks: 2005–2008. *Journal of Clinical Epidemiology*, *63*(11), 1179-1194.
Chung, F., Yegneswaran, B., Liao, P., Chung, S. A., Vairavanathan, S., Islam, S., Khajehdehi, A., & Shapiro, C. M. (2008). STOP questionnaire: A tool to screen patients for obstructive sleep apnea. *Anesthesiology, 108,* 812-921.
De Menses-Gaya, I. C., Zuardi, A. W., Loureiro, S. R., de Souza Crippa, J. A. (2009). Psychometric properties of the Fagerstrom Test for Nicotine Dependence. *Jornal Brasileiro de Pneumologia, 35,* 73-82.
Devilly, G. J., & Borkovec, T. D. (2000). Psychometric properties of the credibility/expectancy questionnaire. *Journal of Behavior Therapy and Experimental Psychiatry, 31*, 73-86.
Ebbert, J. O., Patten, C. A., & Schroeder, D. R. (2006). The Fagerstrom Test for Nicotine Dependence – Smokess Tobacco (FTND-ST). *Addictive Behaviors,31,* 1716-1721.
Ferketich, A. K., Wee, A. G., Schultz, J., & Wewers, M. E. (2007). A measure of nicotine dependence for smokeless tobacco users. *Addictive Behaviors, 9,* 1970-1975.
Flynn, K. E., Lin, L., Cyranowski, J. M., Reeve, B. B., Reese, J. B., Jeffery, D. D., ... & Weinfurt, K. P. (2013). Development of the NIH PROMIS® sexual function and satisfaction measures in patients with cancer. *The Journal of Sexual Medicine*, *10*(S1), 43-52.
Foa, E. B., Elhers, A., Clark, D. M., Tolin, D. F. & Orsillo, S. M. (1999). The Posttraumatic Cognitions Inventory (PCTI). *Psychological Assessments, 11*, 303-314.
Forbes, D., Alkemade, N., Hopcraft, D., Hawthorne, G., O'Halloran, P., Elhai, J. D., … Lewis, V. (2014). Evaluation of the Dimensions of Anger Reactions-5 (DAR-5) scale in combat veterans with posttraumatic stress disorder. *Journal of Anxiety Disorders, 28,* 830-835.
Forbes, D., Hawthorne, G., Elliott, P., McHugh, T., Biddle, D., Creamer, M., & Novaco, R. W. (2004). A concise measure of anger in combat-related posttraumatic stress disorder. *Journal of Traumatic Stress, 17,* 249-256.
Gray, M. J., Litz, B. T., Hsu, J. L., Lombardo, T. W. (2004). Psychometric properties of the Life Events Checklist. *Assessment, 11*, 330-341.
Heatherton, T. F., Kozlowski, L. T., Frecker, R. C., & Fagerstrom, K. O. (1991). The Fagerstrom Test for Nicotine Dependence: A revision of the Fagerstrom Tolerance Questionnaire. *British Journal of Addiction, 86,* 1119-1127.

Jeffery, D. D., Tzeng, J. P., Keefe, F. J., Porter, L. S., Hahn, E. A., Flynn, K. E., ... & Weinfurt, K. P. (2009). Initial report of the cancer Patient-Reported Outcomes Measurement Information System (PROMIS) sexual function committee. *Cancer*, *115*(6), 1142-1153.

Johnson, D. C., Polusny, M. A., Erbes, C. R., King, D., King, L., Litz, B. T., Schnurr, P. P., Friedman, M., Pietrzak, R. H. & Southwick, S. M. (2008). *Resilience and response to stress: Development and initial validation of the Response to Stressful Experiences Scale (RSES).* Unpublished manuscript, Naval Health Research Center, San Diego, CA.

Kazis, L. E., Selim, A., Rogers, W., Ren, X. S., Lee, A., & Miller, D. R. *Veterans RAND 12-Item Health Survey (VR-12): A White Paper Summary*. Unpublished manuscript. http://www.hosonline.org/surveys/hos/download/veterans_rand_12_item_health_survey_white_paper_summary.pdf

Kroenke, K., Spitzer, R. L., & Williams, J. B. W. (2001). The PHQ-9: Validity of a brief depression severity measure. *Journal of General Internal Medicine, 16,* 606-613.

Kroenke, K., Spitzer, R. L., & Williams, J. B. (2002). The PHQ-15: Validity of a new measure for evaluating the severity of somatic symptoms. *Psychosomatic medicine*, *64*, 258-266.

Kroenke, K., Spitzer, R. L., Williams, J. B., & Lowe, B. (2010). The Patient Health Questionnaire Somatic, Anxiety, and Depressive Symptom Scales: A systematic review. *General Hospital Psychiatry, 32,* 345-359.

Kubany, E. S., Haynes, S. N., Abueg, F. R., Manke, F. P., Brennan, J.M., Stahura, C. (1996). Development and validation of the Trauma-Related Guilt Inventory (TRGI). *Psychological Assessments, 8*, 423-444.

Lowe, B., Decker, O., Muller, S., Brahler, E., Schellberg, D., Herzog, W., & Herzberg, P. Y. (2008). Validation and standardization of the Generalized Anxiety Disorder Screener (GAD-7) in the general population. *Medical Care, 46,* 266-274.

Marx, B. P. (2013). *Development and validation of a PTSD-related impairment scale.* Retrieved from www.dtic.mil/cgi-bin/GetTRDoc?AD=ADA585414

McCarroll, J. E., Ursano, R. J., Liu, X., Thayer, L. E., Newby, J. H., Norwood, A. E. & Fullerton, C.S. (2000). Deployment and the probability of spousal aggression by U.S. Army soldiers. *Military Medicine, 165*, 41-44.

McDonald, S. D., & Calhoun, P. S. (2010). The diagnostic accuracy of the PTSD Checklist: A critical review. *Clinical Psychology Review, 30*, 976-987.

Metalsky, G. I., & Joiner, T. E. (1997). The Hopelessness Depression Symptom Questionnaire. *Cognitive Therapy and Research, 21,* 359-384.

Morin, C.M. (1993). *Insomnia: Psychological Assessment and Management.* New York, NY: The Guilford Press.

Nock, M. K., Holmberg, E. B., Photos, V. I., & Michel, B. D. (2007). Self-Injurious Thoughts and Behaviors Interview: Development, reliability, and validity in an adolescent sample. *Psychological Assessment, 19,* 309-317.

Novaco, R. (1975). *Dimensions of anger reactions*. Irvine, CA: University of California.

Novaco, R. W., Swanson, R. D., Gonzalez, O. I., Gahm, G. A., & Reger, M. D. (2012).Anger and postcombat mental health: Validation of a brief anger measure with U.S. soldiers postdeployed from Iraq and Afghanistan. *Psychological Assessment, 24,* 661–675.

Reinert, D. F., & Allen, J. P. (2007). The Alcohol Use Disorders Identification Test: An update of research findings. *Alcoholism: Clinical and Experimental Resesarch, 31,* 185-199.

Roy, M., Dum, M., Sobell, L. C., Sobell, M. B., Simco, E. R., Manor, H., & Palmerio, R. (2008). Comparison of the Quick Drinking Screen and the alcohol Timeline Followback with outpatient alcohol abusers. *Substance Use and Misuse, 43,* 2116-2123.

Saunders, J. B., Aasland, O. G., Babor, T. F., De La Fuente, J. R. & Grant, M. (1993). Development of the Alcohol Use Disorders Identification Test (AUDIT): WHO collaborative project on early detection of persons with harmful alcohol consumption-II. *Addiction, 88,* 791-804.

Schwab, K. A., Baker, G., Ivins, B., Sluss-Tiller, M., Lux, W., & Warden, D. (2006). The Brief Traumatic Brain Injury Screen (BTBIS): Investigating the validity of a self-report instrument for detecting traumatic brain injury (TBI) in troops returning from deployment in Afghanistan and Iraq. [Abstract]. *Neurology, 66*(5)(Supp. 2), A235.

Schwab, K. A., Ivins, B., Cramer, G., Johnson, W., Sluss-Tiller, M., Kiley, K., Lux, W. & Warden, D. (2006). Screening for traumatic brain injury in troops returning from deployment in Afghanistan and Iraq: Initial investigation of the usefulness of a short screening tool for traumatic brain injury. *Journal of Head Trauma Rehabilitation, 22*(6), 377-389.

"Sexual Function and Satisfaction Measures User Manual." (2012). *Assessment Center – PROMIS.* Manual available from https://www.assessmentcenter.net/documents/Sexual%20Function%20Manual.pdf

Sheehan, D.V., Harnett-Sheehan, K., & Raj, B.A. (1996). The measurement of disability. International Clinical Psychopharmacology, 11(suppl 3), 89-95.

Sheehan, D. V., Lecrubier, Y., Sheehan, K. H., Amorim, P., Janavs, J., Weiller, E., …Dunbar, G. C. (1998). The Mini-International Neuropsychiatric Interview (M.I.N.I.): The development and validation of a structured diagnostic psychiatric interview for DSM-IV and ICD-10. *Journal of Clinical Psychiatry, 59,* 22-33.

Sobell, L. C., Agrawal, S., Sobell, M. B., Leo, G. I., Young, L. J., Cunningham, J. A., Simco, E. R. (2003). Comparison of a Quick Drinking Screen with the Timeline Followback for individuals with alcohol problems. *Journal of Studies on Alcohol, 64,* 858-861.

Spitzer, R. L., Kroenke, K., & Williams, J. B. W. (1999). Validation and utility of a self-report version of the PRIME-MD: The PHQ primary care study. *Journal of the American Medical Association, 282*, 1737-1744.
Spitzer, R. L., Kroenke, K., Williams, J. B., & Lowe, B. (2006). A brief measure for assessing generalized anxiety disorder: The GAD-7. *Archives of Internal Medicine, 166,* 1092-1097.
Straus, M. A. (1979). Measuring intrafamility conflict and violence: The Conflict Tactics (CT) Scales. *Journal of Marriage and the Family, 41*, 75-88.
Straus, M. A. & Douglas, E. M. (2004). A short form of the revised conflict tactics scales, an dtypologies for severity and mutuality. *Violence and Victims, 19*(5), 507-520.
Straus, M. A., Hamby, S. L., Boney-McCoy, Sugarman, D. B. (1996). The Revised Conflict Tactics Scales (CTS2): Development and preliminary psychometric data. *Journal of Family Issues, 17*(3), 283-316.
Taft, C.T., Street, A.E., Marshall, A.D., Dowdall, D.J., & Riggs, D.S. (2007). Posttraumatic stress disorder, anger, and partner abuse among Vietnam combat veterans. *Journal of Family Psychology, 21,* 270-277.
Vogt, D. S., Smith, B. N., King, L. A., King, D. W., Knight, J. A., & Vasterling, J. J. (2013). Deployment Risk and Resilience Inventory-2 (DRRI-2): An updated tool for assessing psychosocial risk and resilience factors among service members and veterans. *Journal of Traumatic Stress, 26*, 710-717.
Weathers, F.W., Blake, D.D., Schnurr, P.P., Kaloupek, D.G., Marx, B.P., & Keane, T.M. (2013). *The Clinician-Administered PTSD Scale for DSM-5 (CAPS-5).* Interview available from the National Center for PTSD at www.ptsd.va.gov.
Weathers, F. W., Blake, D. D., Schnurr, P. P., Kaloupek, D. G., Marx, B. P., & Keane, T. M. (2013). *The Life Events Checklist for DSM-5 (LEC-5).* Instrument available from the National Center for PTSD at www.ptsd.va.gov.
Weathers, F. W., Keane, T. M., & Davidson, J. R. (2001). Clinician-administered PTSD scale: A review of the first ten years of research. *Depression and Anxiety*, *13*, 132–156.
Weathers, F. W., Litz, B. T., Herman, D. S., Huska, J. A., & Keane, T. M. (1993). *The PTSD Checklist (PCL): Reliability, validity, and diagnostic utility.* Paper presented at the 9th Annual Conference of the ISTSS, San Antonio, TX.
Weathers, F. W., Litz, B. T., Keane, T. M., Palmieri, P. A., Marx, B. P., & Schnurr, P. P. (2013). *The PTSD Checklist for DSM-5 (PCL-5).* Instrument available from the National Center for PTSD at www.ptsd.va.gov.
Weinfurt, K. P., Lin, L., Bruner, D. W., Cyranowski, J. M., Dombeck, C. B., Hahn, E. A., … Flynn, K. E. (2015). Development and initial validation of the PROMIS Sexual Function and Satisfaction Measures Version 2.0. *Journal of Sexual Medicine, 12,* 1961-1974.
Yu, L., Buysse, D. J., & Germain, A. (2012). Development of short forms from the PROMIS Sleep Disturbance and Sleep-Related Impairment item banks. *Behavioral Sleep Medicine, 10,* 6-24.

13.0 TIME REQUIRED TO COMPLETE THE RESEARCH (including data analysis). 3 years

14.0 STUDY CLOSURE PROCEDURES. At the conclusion of the study (following completion of manuscripts) or termination by either the Investigators or the IRB, all data not included in the STRONG STAR Repository (BAMC IRB, C.2011.054d; UTHSCSA IRB, HSC20100475H) will be stripped of identifiers. De-identified (anonymized) data will be maintained indefinitely. Informed consent documents will be stored securely for a minimum of three years following completion of the research; HIPAA authorizations will be stored for a minimum of six years IAW Federal regulations. A protocol completion form will be filed with the IRB.

15.0 Funding: This research is supported by Consortium to Alleviate PTSD (CAP) award numbers W81XWH-13-2-0065 from the U.S. Department of Defense, Defense Health Program, Psychological Health and Traumatic Brain Injury Research Program (PH/TBI RP), and I01CX001136-01 from the U.S. Department of Veterans Affairs, Office of Research & Development, Clinical Science Research & Development Service under the title, "Project Remission: Maximizing Outcomes with Intensive Treatments for PTSD" (Project PI: Alan Peterson, Ph.D.). Texas Health and Human Services is also providing funding to pay for the travel costs of Texas veteran participants who live outside the local area.

16.0 Description of Assessments: The majority of the measures listed below is commonly used, have adequate to good psychometrics, and are part of the Consortium common data elements (CDE). As outlined in the National Research Action Plan, evidence-based CDEs and measures for CAP studies will ensure comparability of results across the consortium as well as other clinical trials and epidemiological studies of PTSD. Please see below and **Study Procedures/Research Interventions** for administration schedule.

1. <u>The Clinician Administered PTSD Scale for DSM-5 (CAPS-5; Weathers, Blake, Schnurr, Kaloupek, Marx, & Keane, 2012):</u> The CAPS-5 is structured interview that assesses the DSM-5 criteria for PTSD (Weathers et al., 2013). Each item is rated on a severity scale ranging from 0 (Absent) to 4 (Extreme/incapacitating) and combines information about frequency and intensity for each of the 20 symptoms. Additional items that are not included in the total score

evaluate overall symptom duration, distress, impairment, dissociative symptoms, and global ratings by the interviewer. Validation studies are nearly complete to establish the psychometric properties of the CAPS-5 and findings will be reported in peer-reviewed publications. This interview is very similar to its predecessor, the CAPS for DSM-IV, which has been considered the gold standard for evaluating PTSD and demonstrated good reliability and validity (Weathers, Keane, & Davidson, 2001). In addition to reflecting diagnostic changes for PTSD in DSM-5, the CAPS-5 differs from the CAPS in that frequency and intensity ratings for each symptom are no longer scored separately, so the severity rating for each item determines whether a symptom is present or not. Subscale scores are calculated by summing severity scores for items in the following PTSD symptom clusters: re-experiencing, avoidance, negative alterations in cognitions and mood, and hyperarousal. Scores ≥ 25 indicate a probable diagnosis of PTSD.

2. PTSD CheckList-5 (PCL-5): The PCL-5 (Weathers, et al., 2010) is a 20-item self-report measure update of the PCL designed to assess PSTSD symptoms as defined by the DSM-5. The PCL-5 is currently available and has been shown to have good psychometric properties. The PCL-5 evaluates how much participants have been bothered by PTSD symptoms in the past month (for baseline and follow up assessments) or the past week (all interim assessments) as a result of a specific life event. Each item of the PCL-5 is scored on a five point scale ranging from 0 “not at all”) to 4 (“extremely).

3. Sheehan Disability Scale: The SDS is a 3 item self-report measure designed to measure the extent to which three areas (work, social life, and home life/family responsibilities) in the individual’s life are compromised by panic, anxiety, phobic, or depressive symptoms. The SDS is not to be used for diagnostic purposes, however, it may be used to supplement a mental health assessment by providing a summary of functional impairment.

4. Brief Inventory of Psychosocial Functioning: The Brief Inventory of Psychosocial Functioning (Marx et al. 2013) is a 7-item self-report instrument measuring respondents’ level of functioning in seven life domains: romantic relationship, relationship with children, family relationships, friendships and socializing, work, training and education, and activities of daily living. Respondents indicate the degree to which they had trouble in the last 30 days in each area on a 7-point scale ranging from “0 = Not at all” to “6 = Very much.”

5. Demographics and Military Service Characteristics Form: The Demographics Form measures standard demographics (race, gender, age) and military service information (e.g., rank).

6. Combat-Related PTSD Beliefs Inventory: The Combat-Related PTSD Beliefs Inventory is being added to Project Remission study as a self-report measure of participant’s general beliefs about combat-related PTSD. On each of the four items, participants will be asked to rate the degree to which they agree or disagree with the statements on a 1 to 10 scale with 1 = Strongly Disagree and 10 = Strongly Agree. The measure was created specifically for Project Remission. Therefore, there are no validity or reliability data on the measure. The results of each item will be compared with posttreatment change scores on the PCL-5 and CAPS-5 to determine if they are correlated with treatment response.

7. History of Head Injuries: The History of Head Injuries form was developed from the Defense and Veterans Brain Injury Center (DVBIC) 3-Item Screening Tool (Schwab, Baker, Ivins, Sluss-Tiller, Lux & Warden, 2006; Schwab, Ivins, Cramer, Johnson, Sluss-Tiller, Kiley, Lux & Warden, 2006). The DVBIC Screening Tool, initially called the Brief Traumatic Brain Injury Screen (BTBIS), was used as the gold standard for the diagnosis of TBI in a sample of soldiers returning from duty in Iraq and/or Afghanistan (Schwab, Ivins, et al., 2006). As recommended by the DVBIC, the 3-Question Screen will be considered positive when the participant endorses an injury (question 1) and altered consciousness (question 2, items A-E) for the worst head injury sustained while deployed. The form was modified for STRONG STAR and now CAP to capture the number of injuries, and to answer question 2 based on the worst injury; the original form does not recognize the possibility of multiple head injuries during deployment. As the 3-Question Screen does not query head injuries prior to deployment, an additional four questions have been added to solicit information about each head injury sustained outside of deployment.

8. Health Interview. The original Health Care Utilization (HCU) is a 16-item questionnaire developed in 2000 for Dr. Patricia A. Resick’s NIH grant, “Cognitive Processes in PTSD: Treatment II.” The questionnaire was based on the 1999 Behavioral Risk Factor Surveillance System. The version that will be administered as part of the STRONG STAR Consortium has been modified to be of increased relevance to active-duty service personnel. The measure includes items regarding use of mental health services, current psychiatric medication, past psychiatric medication, hospitalization, and outpatient medical services, as well as items intended to assess changes in participants’ military status. Tobacco/nicotine use will also be queried.

9. Mini-International Neuropsychiatric Interview (MINI-7.0). The MINI 7.0 is a short, structured clinical diagnostic interview designed to cover the major psychiatric disorders in DSM-5 and ICD-10. It is widely used in epidemiological studies and multi-site clinical trials. Responses to the interviewer's questions are rated as either "yes" or "no." As is the case on the SCID, there are skip-outs, which saves time. However, this means that the MINI cannot be used to index the severity of a given psychiatric problem, only caseness. When there are many skip-outs, the MINI takes ~15 minutes to administer. The MINI can be used to assess the full spectrum of psychiatric problems, or specific modules can be employed (e.g., the schizophrenia module to rule out thought disorder).

10. Alcohol Use Disorders Identification Test (AUDIT): The AUDIT (Babor et al, 2001) will be used to identify people with hazardous or harmful patterns of alcohol consumption. The AUDIT is a 10-item screening measure, developed by the World Health Organization (WHO), with three subscales (alcohol consumption, drinking behavior, and alcohol-related problems) that are scored on a 4-point scale for a highest possible total score of 40. Among those identified as using alcohol in a harmful manner, 92% had scores of 8 or more, though determining a cutoff score should be left up to the clinician, depending upon the population being studied. The AUDIT has good internal consistency (α = .80-.93) as well as sensitivity and specificity (Saunders, Aasland, Babor, De La Fuente & Grant, 1993).

11. Deployment Risk and Resilience Inventory-2 (DRRI-2) Combat Experience and Post-battle -Scales: High- and low-intensity deployment stress exposure will be assessed using scales from the DRRI-2 (Vogt, Smith, King, & King, 2012). The DRRI-2 is an update of the original DRRI (King, King, Vogt, Knight, & Samper, 2006), which was developed and tested in three separate national samples of veterans of the first Gulf War. It has been revised and tested with OEF/OIF/OND returnees (Vogt et al., 2008). The DRRI-2 provides an update of the DRRI's assessment of deployment-related factors to ensure the instrument's applicability across a variety of deployment circumstances (e.g., different eras of service) and military subgroups (e.g., men and women), as well as to validate updated measures in a contemporary Veteran cohort (Vogt, et al., 2012). High intensity stressor exposures will be assessed using the DRRI Combat Experiences and Aftermath of Battle subscales. Responses to these scales are on a 6-point Likert scale. The total score is the sum of the item scores, where higher scores signify greater exposure to combat or exposure to the consequences of combat, respectively. Both subscales have very good internal consistency (α = .90 to .92) and construct validity (Vogt et al., 2012).

12. Depressive Symptoms Index-Suicidality Subscale (DSI-SS): The DSI-SS (Metalsky & Joiner, 1997) will be used to assess current suicidal ideation. The DSI-SS is a 4-item self-report measure of suicidal ideation that focuses on ideation, plans, perceived control over ideation, and impulses for suicide. It is being used as a core measure in the Military Suicide Research Consortium. Scores on each item range from 0 to 3, with higher scores reflecting greater severity of suicidal ideation. Instructions will instruct the participants to respond based on the past two weeks (for baseline and follow up visits) or the past week (for interim assessment visits). A systematic review of measures of suicidal ideation and behaviors found that the DSI-SS had evidence of excellent internal consistency and concurrent validity (Batterham et al., 2014).

13. Self-Injurious Thoughts and Behaviors Interview (SITBI): The SITBI (Nock, Holmberg, Photos, & Michel, 2007) is a structured interview assessing the presence, frequency, and characteristics of self-injurious and suicidal thoughts and behaviors. The SITBI will be administered by an Independent Evaluator, who will instruct the participants to answer the questions based on their entire lifetime of experience. The SITBI has shown high interrater reliability, test-retest reliability, and concurrent validity (Nock et al., 2007).

14. Patient Health Questionnaire-9 (PHQ-9): The PHQ-9 is a widely used and well-validated instrument for measuring the severity of depressive symptoms (Kroenke, Spitzer, & Williams, 2001). It consists of 9 items that assess both affective and somatic symptoms related to depression and depressive disorders; these 9 items correspond to the diagnostic criteria for DSM MDD. Respondents rate the frequency with which they have been bothered by depressive symptoms within the past two weeks (for baseline and follow up visits) or the past week (for interim assessment visits)on a scale ranging from 0 ("not at all") to 3 ("nearly every day"). Scores on all items are summed to obtain a total severity score. Scores reflect no significant depressive symptoms (0-4), mild depressive symptoms (5-9), moderate depressive symptoms (10-14), moderately severe depressive symptoms (15-19), and severe depressive symptoms (>19). Respondents also indicate the degree to which their depressive symptoms have made it difficult for them to do their work, take care of things at home, or get along with other people, from "not difficult at all" to "extremely difficult." The PHQ-9 has high internal consistency (e.g., alpha ranging from .83 to .92; Cameron, Crawford, Lawton, & Reid, 2008), and correlates strongly with other measures of depression (Kroenke et al., 2001).

15. Generalized Anxiety Disorder Screener (GAD-7): The GAD-7 (Spitzer, Kroenke, Williams, & Lowe, 2006) will be used to assess generalized anxiety symptomology. This is a 7-item measure that asks participants to rate the frequency

with which they have been bothered by anxiety symptoms within the past two weeks on a scale ranging from 0 (“not at all”) to 3 (“nearly every day”). Scores on all items are summed to obtain a total severity score. Scores reflect no significant anxiety symptoms (0-4), mild anxiety symptoms (5-9), moderate anxiety symptoms (10-14), and severe anxiety symptoms (>15). Respondents also indicate the degree to which their anxious symptoms have made it difficult for them to do their work, take care of things at home, or get along with other people, from “not difficult at all” to “extremely difficult.” The GAD-7 has been shown to have high internal consistency (e.g., $\alpha = .89$; Lowe et al., 2008) and has been shown to reliably discriminate between anxious and non-anxious diagnostic groups (Kroenke, Spitzer, Williams, & Lowe, 2010).

16. <u>Veterans Rand 12-Item Health Survey (VR-12)</u>: Because a certain level of PTSD symptoms is an occupational hazard among service members redeployed for combat, it is critical to pay close attention to functional capacities as an important index of intervention efficacy. The Veterans SF-36 (VR-36) was adapted from the RAND SF-36 Version 1.0 questionnaire, and spans the range of health domains from physical to psychological health status. It includes two modifications. The first modification is an increase in the number of response choices for the role physical (RP) and role emotional (RE) items from a two point yes/no choice to a five-point likert scale (no, none of the time, yes, a little of the time, yes, some of the time, yes, most of the time, yes, all of the time). The second modification is the use of two items to assess health change, one focusing on physical health and one on emotional problems, in contrast to the one general change item in the RAND SF-36 (Kazis, Lee et al., 2004; Kazis, Miller, Clark et al 2004). The VR-36 has been widely used, distributed and documented in the Veterans Health Administration (VHA) with close to 2 million questionnaires administered nationally in six national surveys since 1996. The changes to the survey have increased the overall precision of the instrument and the discriminant validity of the physical and mental component summary scales (Kazis, Nethercot, et al 2006). The VR-36 is comprised of 37 items and eight scales: physical functioning, role limitations due to physical problems, bodily pain, general health perceptions, energy/ vitality, social functioning, role limitations due to emotional problems, and mental health. Also, there are two summary scales: a physical component summary (PCS) and mental component summary (MCS). Higher scores indicate better health. Each summary is expressed as a T score, which facilitates comparisons between the VA patients and the general U.S. population. The PCS and MCS scores provide at least 90% of the reliable variance in the eight SF-36 concepts (Kazis & Wilson, 1998; Kazis, Wilson, et al., 1999). The Veterans SF-12 was developed from the Veterans SF-36 and adapted from the MOS SF-36. It includes fewer items for seven of the eight scales and provides 90% of the reliable variance in the two component summary measures using the Veterans SF-36. Using independent results from the Veterans Health Study and the 1996 National Survey of Ambulatory Care Patients, the results for the Veterans SF-12 corresponded very closely with the results for the Veterans SF-36 (average differences of 0.06 points between them for PCS and 0.31 points for MCS; Kazis et al., 1996; Kazis & Wilson, 1998).

17. The Dimensions of Anger Reactions-5 (DAR-5). The DAR-5 (Forbes et al., 2014) is a short form version of the original Dimensions of Anger Reactions (Novaco, 1975). It addresses anger frequency, intensity, duration, aggression, and interference with social functioning. Respondents indicate the degree to which each of 5 items describes their feelings or behavior over the last 4 weeks, from 1 (“none or almost none of the time”) to 5 (“all or almost all of the time”). The items are summed for a score ranging from 5 to 25; a cut-point of 12 is recommended to indicate a level of anger that may warrant clinical attention (Forbes et al., 2014). The original DAR has shown good psychometric properties in samples of Vietnam veterans (Forbes et al., 2004) and treatment-seeking Iraq and Afghanistan veterans (Novaco, Swanson, Gonzalez, Gahm, & Reger, 2012). In a sample of veterans across eras who were diagnosed with combat-related PTSD (Forbes et al., 2014), the DAR-5 showed good internal consistency ($\alpha = .86$), convergent validity with the STAXI, and concurrent and discriminant validity. The DAR has also shown sensitivity to change following PTSD treatment (Forbes et al., 2004*)*.

18. <u>Conflict Tactics Scale (revised) (CTS2) Physical Assault & Psychological Aggression Subscales</u>: The CTS was designed to assess the use of reasoning, verbal aggression, and violence within the family (Straus, 1979) and over time has become the most widely used instrument to assess intimate partner violence (Straus & Douglas, 2004; Straus, Hamby, Boney-McCoy & Sugarman, 1996). The CTS (revised; CTS2) poses 39 questions to assess five tactics used when there is conflict in the relationships of dating, cohabitating, or marital couples, i. e., Physical Assault, Psychological Aggression, Negotiation, Injury, and Sexual Coercion (Straus, Hamby, Boney-McCoy & Sugarman, 1996). In 2004 a revised Conflict Tactics Scales (CTS2S) was developed shortening the instrument from 78 questions to 20 reducing the test administration time to three minutes (Straus & Douglas, 2004). For the STRONG STAR studies, only the Physical Assault and Psychological Aggression subscales (20 items) of the CTS2 will be used broadening the assessment of conflict to query interpersonal conflict among friends, colleagues, and acquaintances as well as the family as these subscales represent the content areas of most interest and are most relevant to the targeted populations. Testing in a sample of 317 undergraduate students from well-educated parents, the CTS2

demonstrated alpha reliabilities ranging from 0.79 to 0.95 (Straus, Hamby, Boney-McCoy & Sugarman, 1996). Furthermore, Straus et al (1996) felt that the instrument demonstrated construct and discriminate validity in that men were shown to be more likely to use coercion to obtain sex and more likely to have serious injury result from physical assault. Discriminate validity was demonstrated in that two of the subscales, negotiation and sexual coercion and negotiation and injury, were shown to not be correlated. Various versions of the CTS have been used in other studies of military personnel (Adler et al, 2008; McCarroll et al., 2000; Taft, Street, Marshall, Dowdall, & Riggs, 2007), but to assess conflict in the family and not wider affiliations as STRONG STAR intends to assess.

19. Quick Drinking Screen (QDS) self-report version: The QDS (Sobell et al., 2003) will be used to measure alcohol consumption. It consists of 4 items probing frequency and quantity of alcohol consumption. It will be administered in a self-report form. The QDS has been validated against the Timeline Followback daily estimation measure of alcohol use, and it shows good psychometric properties (Roy et al., 2008; Sobell et al., 2003). The QDS's time-frame will be modified to match the "last two weeks" probed by the mandated depression and anxiety instruments for CAP studies (PHQ-9 and GAD-7). Like these other measures, the QDS can be administered frequently throughout CAP trials to track changes in alcohol use.

20. Response to Stressful Experiences Scale (RSES). The RSES is a 22-item questionnaire developed by a team of experts at the National Center for PTSD to assess trait-related cognitive, emotional, and behavioral resilience (Johnson, et al., 2008). It asks participants to assess how well each statement describes them, both during and after stressful events in their lives. Responses are given on a 5-point scale, with anchors 0 (not at all like me) to 4 (exactly like me). Psychometric testing in 1,014 active duty, reserve and veteran groups showed that the instrument has sound internal consistency (coefficient alpha 0.91 to 0.93) as well as good test-retest reliability over 7-days (reliability correlation = 0.87). The instrument correlated positively with another measure of resilience, the Connor-Davidson Resilience Scale (coefficient alpha 0.61 to 0.81) as well as unit cohesion (coefficient = 0.38), and post-deployment support (coefficient 0.36 to 0.56). The instrument correlated negatively with psychological symptom distress as assessed with the Patient Health Questionnaire - 9 (coefficient = -0.51), posttraumatic stress as assessed with the PCL-M (coefficient -0.23 to -0.39), and overall mental health as assessed with the Minnesota Multiphasic Personality Inventory-2 Neuroticism (coefficient = -0.35) demonstrating concurrent validity. Factor analysis revealed a six-factor model of resilience including subscales for active coping, meaning-making, cognitive flexibility, spirituality, self-efficacy, and restoration.

21. Life Events Checklist-5 (LEC-5). The LEC includes a list of 24 potentially traumatic life events commonly associated with PTSD symptoms. The instrument was designed to facilitate the diagnosis of PTSD (Weathers, Blake, Schnurr, Kaloupek, Marx, & Keane, 2013a).). In this study, the LEC-5 will also be used to identify the index event and focus of the PTSD treatment. For each potentially traumatic life event, respondents rate their experience of that event on a 5-point nominal scale (1 = happened to me, 2 = witnessed it, 3 = learned about it, 4 =part of my job, 5= not sure, and 6 = does not apply). Each nominal point will be scored separately, as either 0 (=not endorsed by participant) or 1 (=endorsed by participant).

22. PTSD Diagnostic Scale for DSM-5 (PDS-5): The PDS is a 49-item self-report measure recommended for use in clinical or research settings to measure severity of PTSD symptoms related to a single identified traumatic event. The PDS is unique in that it assesses all of the DSM criteria for PTSD and inquires about the past month (time frame can be adjusted for different uses). The PDS has been shown to be a reliable measure with excellent internal consistency overall (alpha = .92) and very good internal consistency for the symptom subscales (alphas ranging from .78 to .84; Orsillo, 2001). Additionally, repeated administration over 2 to 3 weeks yielded an 87% agreement rate (kappa =.74) between diagnoses and adequate stability in symptom severity (all *r*'s .77 to .85). The PDS has also been demonstrated to be a valid measure, with satisfactory agreement between the diagnoses derived from the PDS and those obtained from a structured clinical interview (kappa of .65, 82% agreement). Sensitivity of the PDS was .89 and specificity was .75. Scores reflecting symptom severity on the PDS correlated with another measure of PTSD (r = .78), a measure of anxiety (*r*'s range from .73 to .74), and a measure of depression (r =.79). Edna Foa, PhD, is the copyright owner for the PDS-5. She is also a co-principal investigator for Project Remission, and she has approved the in-kind use of this measure without cost.

23. Deployment Risk and Resiliency Inventory-2 (DRRI-2) Post-Deployment Social Support subscale: The DRRI-2 (Vogt et al., 2012) is a suite of 17 individual scales that assess key deployment-related risk and resilience factors with demonstrated implications for veterans' long-term health. The Post-Deployment Social Support subscale will be used to assess post-deployment social support as a factor that may affect recovery.

24. Patient Health Questionnaire-15 (PHQ-15; Kroenke, Spitzer, & Williams, 2002). The PHQ-15 is a brief, self-administered

questionnaire that assesses somatic symptom severity. Participants rate the severity of 15 somatic symptoms as 0 (not bothered at all), 1 (bothered a little) or 2 (bothered a lot). The scale has strong psychometric properties in terms of internal reliability, convergent validity, and discriminant validity (Kroenke, et al., 2002), and has been used in recent research using an active duty military sample (Hoge, et al., 2008).

25. Trauma Related Guilt Inventory (TRGI) (brief): The TRGI was developed to assess guilt feelings and attitudes about a specific traumatic event (Kubany, Haynes, Abueg, Manke, Brennan, Stahura, 1996). Often survivors of trauma experience guilt related to the trauma about things they did or did not do or feelings they had or did not have. A combat veteran may experience guilt about having provided first aid to some of his or her wounded colleagues but not others even though it was not possible to care for everyone. Or, an individual may experience survivor's guilt not understanding why he lived while others died. These feelings of guilt can be important in evaluating the various treatments for PTSD. The TRGI is scored into three scales (i. e., 4-item Global Guilt Scale, 6-item Distress Scale, and a 22-item Guilt Cognitions Scale) and 3 subscales (i. e., the Hindsight-Bias / Responsibility Subscale, the Wrongdoing Subscale, and the Lack of Justification Subscale). Psychometric testing has been conducted using almost 600 individuals including 357 university students, 163 women receiving counseling services in a battered women's program, and 74 Vietnam veterans. Internal consistency was high across all the testing samples. In the sample of Vietnam veterans the alpha coefficient ranged from 0.66 to 0.94. In the Vietnam veterans, the scores on the various scales and subscales were significantly correlated with the Posttraumatic CheckList – Military (PCL-M), the Mississippi Scale for Combat-Related Posttraumatic Stress Disorder, the Zung Self-Rating Depression Scale, the Guilt Inventory, and the Social Avoidance and Distress Scale with reliability coefficients ranging from 0.36 to 0.77 ($p<.05$). In a sample of 32 university students, the test-retest correlations after two days ranged from 0.74 to 0.83. An abbreviated 16-item version of the TRGI will be used in the STRONG STAR studies allowing only for the calculation of the three subscale scores. The Hindsight-Bias / Responsibility Subscale score = (sum of scores on Items 1, 5, 9, 14, 19, 23, and 26) divided by 7. The Wrongdoing Subscale score = (sum of scores on Items 3, 7, 11, 16, and 21) divided by 5. And, the Lack of Justification Subscale score = [sum of scores on Items 4 (R), 8 (R), 12 (R), and 17 (R)] divided by 4.

26. Posttraumatic Cognitions Inventory (PTCI): The PTCI is a 36-item questionnaire that was developed to determine how an individual views the trauma and its sequelae in an attempt to understand both how PTSD develops and is maintained (Foa, Elhers, Clark, Tolin, & Orsillo, 1999). Using an emotional processing theory, Foa and her colleagues (1999) have suggested that PTSD is a consequence of disruptions in the normal processes of recovery when an individual has excessively rigid concepts about self and world rendering the person vulnerable if a traumatic event occurs. Thus the PTCI was developed as a measure of trauma-related thoughts and beliefs. It is comprised of three subscales (Negative Cognitions about the Self, Negative Cognitions about the World, and Self-Blame). The measure was tested in almost 600 adult volunteers recruited from two university PTSD treatment clinics as well as a university community. Approximately 65% (n=392) of individuals reported having experienced a trauma in which their own life or that of another person was perceived to be in danger and their response at the time included intense terror, horror, or helplessness (Criterion A event). The remaining 35% (n=162) denied such a traumatic experience. Of those who had experienced a trauma, 170 had PTSD symptoms of at least moderate severity while the remaining 185 reported a low symptom severity. The three subscales of the PTCI demonstrated internal consistency with alpha coefficients ranging from 0.86 to 0.97. Convergent validity was demonstrated comparing the PTCI to appropriate subscales of the World Assumptions Scale and Personal Beliefs and Reactions Scale. Significant correlations between the appropriate subscales ranged from 0.20 to 0.85. The PTCI was able to differentiate individuals with and without PTSD demonstrating discriminate validity (sensitivity = 0.78, specificity = 0.93). Test–retest reliability for each of the three subscales at a 1-week interval ranged from 0.75 to 0.89 and for a 3-week interval ranged from 0.80 to 0.86.

27. Childhood Trauma Questionnaire (CTQ): (Bernstein and Fink 1998) The CTQ is 28-item self-report screening for childhood trauma. This measure is included because childhood trauma history has been found to influence neuroendocrine and neurosteroid function beyond what is explained by PTSD (Van Voorhees, Dennis et al. 2014). The CTQ has good convergent validity, internal consistency (.66-.92) and test-retest reliability (.79 -.86) (Bernstein and Fink 1998).

28. Insomnia Severity Index (ISI). The ISI (Morin, 1993) is a 7-item self-report measure that assesses perceived severity of insomnia. Each item uses a 4-point Likert type scale from 0 (not at all satisfied) to 4 (very much satisfied). The items sum to produce a total score (range 0 – 28). The ISI has an internal consistency alpha coefficient of 0.74, and has shown convergent validity with other measures such as the Pittsburgh Sleep Quality Index (r = 0.67), the Dysfunctional Beliefs and Attitudes about Sleep (r = 0.55), and sleep diaries (r ranges from 0.32-0.91) (Bastien, Vallieres & Morin, 2001).

29. PROMIS Sexual Function and Satisfaction Measures: The PROMIS questionnaires are a system of reliable measures of patient–reported health status for physical, mental, and social well–being developed with funding under the National Institutes of Health (NIH) Roadmap for Medical Research Initiative to re-engineer the clinical research enterprise (http://www.nihroadmap.nih.gov). PROMIS measures can be administered across a wide variety of chronic diseases and conditions and in the general population (Cella et al, 2010). One of the PROMIS instruments is a Sexual Functioning Inventory (Jeffery et al, 2009), a revised version of which was recently published (PROMIS Sexual Function and Satisfaction Measures version 2.0; Weinfurt et al., 2015). The PROMIS Sexual Function and Satisfaction Measures (Weinfurt et al., 2015) provide scores on 11 different sub-domains of sexual function: interest in sexual activity, lubrication (female only), vaginal discomfort (female only), clitoral discomfort (female only), labial discomfort (female only), erectile function (male only), orgasm ability, orgasm pleasure, oral dryness, oral discomfort, satisfaction. Each question asks respondents to report on their experiences over the past 30 days. All sub-domain scores are expressed as T scores (mean = 50, standard deviation = 10). In testing with a nationally representative sample of 3516 U.S. adults, correlations between the PROMIS Sexual Function and Satisfaction Measures and corresponding sub-domains of two well-established measures, the Female Sexual Function Index (FSFI) and the International Index of Erectile Function (IIEF), ranged between .67 and .94. The sub-domains of the instrument discriminate between people who had and had not asked a provider about sexual problems. Test-retest correlations over one month are >.65 for all sub-domains (“Sexual Function and Satisfaction Measures User Manual,” 2012).

30. PROMIS Sleep Disturbance and Sleep-Related Impairment short forms: The PROMIS Sleep Disturbance and Sleep-Related Impairment short forms (Yu, Buysse, & Germain, 2012) are self-report measures of past-week sleep disturbance and past-week sleep-related impairment, respectively, derived from the larger PROMIS item banks (Buysse et al., 2010). Each short-form measure includes 8 items, with most items (symptoms) scored in intensity from 1 (“not at all”) to 5 “(very much”). Each measure has shown strong reliability and construct validity (Yu et al., 2012).

31. Snoring, Tired, Observed, Blood Pressure (STOP) Sleep Apnea Screen: To better understand sleep disturbance associated with PTSD and PTSD treatment, the STOP screen (Chung et al., 2008) will be administered to screen for sleep apnea. The STOP is a four-item questionnaire developed and validated in 211 pre-operative surgical patients. Based on the endorsement of 2 or more questions, the sensitivity of the STOP ranged from 66% to 80% as compared with the apnea-hypopnea index (AHI) of polysomnography depending upon the AHI cut-off used. Individuals answering “yes” to 2 or more of the questions will be advised that they may be at risk for having sleep apnea and advised that they may want to speak with their primary care provider to consider referral for an overnight sleep evaluation

32. Fagerstrom Test for Nicotine Dependence (FTND): The FTND (Heatherton et al., 1991) is a 6-item self-report measure that assesses severity of nicotine dependence. Questions probe both quantity of nicotine use (e.g., number of cigarettes per day) and pattern of use (e.g., time to first cigarette in morning). Respondents choose among response options, each of which is assigned a numerical value, with higher numbers corresponding to greater nicotine dependence. Scores on all items are summed to create a severity index (0-2 = very low dependence; 3-4 = low dependence; 5 = medium dependence; 6-7 = high dependence; 8-10 = very high dependence). The Fagerstrom scale has been shown to have high convergent validity with biochemical indices of nicotine use, and the measure has shown acceptable internal consistency (Heatherton et al., 1991). A review of 26 studies of the psychometric characteristics of the Fagerstrom found that it is a reliable instrument for measuring nicotine dependence in diverse settings and populations (Meneses-Gaya et al., 2009).

33. Fagerstrom Test for Nicotine Dependence – Smokeless Tobacco (FTND-ST): This is a modified version of the Fagerstrom Test that focuses on smokeless tobacco use, whereas the original Fagerstrom focuses exclusively on smoking. Like the FTND, the FTND-ST is a 6-item self-report measure of severity of nicotine dependence that has demonstrated convergent validity with biochemical indices of nicotine use (Ebbert et al., 2006; Ferketich et al., 2007). As on the original FTND, respondents choose among response options, each of which is assigned a numerical value, with higher numbers corresponding to greater nicotine dependence. Scores on all items are summed to create a severity index (range = 0–10).

34. Credibility/ Expectancy Questionnaire (CEQ): The CEQ is a 6-item measure that was designed to assess treatment expectancy and rationale credibility for use in clinical outcomes studies (Devilly & Borkovec, 2000). It has been expanded from a 5-item measure designed primarily to assess credibility (Borkovec & Nau, 1972), 4-items of which have been used by both Foa and Resick (P.A. Resick, personal communication, February 22, 2010; E.A. Hembree, personal communication, February 23, 2010; E. B. Foa, personal communication, February 25, 2010) with the name Expectancy of Therapeutic Outcomes (ETO). The 6-item CEQ assesses both whether the person cognitively understands how the therapy works (credibility) as well as whether the person affectively believes that the therapy will work for them personally (expectancy). The 6-item CEQ has been tested in 217 individuals including 68 male Vietnam veterans and 58 female

spouses, 69 individuals diagnosed with general anxiety disorder who had received treatment, and 22 individuals who had received either Cognitive Based Therapy (CBT) or Eye Movement Desensitization and Reprocessing (EMDR) for the treatment of PTSD. The scale demonstrated high internal consistency (alpha coefficients ranged from 0.84 to 0.85). Test-retest reliability over a one-week period was found to be 0.82 for expectancy and 0.75 for credibility. The CEQ was able to differentiate between two treatment rationales in one study, one with and one without an encompassing theory while maintaining equivalence between three rationales in another study. Responses to four questions are scored using a 9-point Likert scale (1= not at all, 9= extremely). Responses to two of the questions are scored using an 11-point Likert Scale (0% to 100%). The combined responses are used to generate a score for credibility and another score for expectancy.

35. The Independent Evaluator Blind Form measures the independent evaluator's best guess about the study treatment that the subject received, level of confidence in this guess, and how unblinding could potentially have occurred. This form will be used at treatment completion and follow-up for all CAP psychotherapy RCTs that include independent evaluation of outcomes.

36. Genetic Biomarkers. Four tubes of blood will be collected and processed for DNA, RNA, plasma, and serum processing.

Appendices:

A – Treatment Manuals:
- Massed-PE
- IOP-PE

B – Recruitment Flyer

C – Telephone Script and Pre-Screen Questionnaire
