# Supplementary material for: Massed vs Intensive Outpatient Prolonged Exposure for Combat-Related Posttraumatic Stress Disorder: A Randomized Clinical Trial
Source: JAMA Netw Open. 2023 Jan 5;6(1):e2249422. doi: 10.1001/jamanetworkopen.2022.49422 (PMC9856757; doi:10.1001/jamanetworkopen.2022.49422)
Supplement: Supplement 2. — eTable 1. Intensive Outpatient Program Prolonged Exposure (IOP-PE) Treatment Augmentations eTable 2. Description of Study Measures eTable 3. Summary of Adverse Events (AEs) Reported During Treatment eReferences. [file jamanetwopen-e2249422-s002.pdf]

Supplemental Online Content

Peterson AL, Blount TH, Foa EB, et al; Consortium to Alleviate PTSD. Massed vs intensive outpatient prolonged exposure for combat-related posttraumatic stress disorder: a randomized clinical trial. *JAMA Netw Open*. 2023;6(1):e2249422. doi:10.1001/jamanetworkopen.2022.49422

eTable 1. Intensive Outpatient Program Prolonged Exposure (IOP-PE) Treatment Augmentations
eTable 2. Description of Study Measures
eTable 3. Summary of Adverse Events (AEs) Reported During Treatment
eReferences

This supplemental material has been provided by the authors to give readers additional information about their work.

© 2023 Peterson AL et al. *JAMA Network Open.*

eTable 1. Intensive Outpatient Program Prolonged Exposure (IOP-PE) Treatment Augmentations

Augmentation	Description	Rationale
1. Team-Based Treatment	In addition to the primary therapist, the treatment team includes a secondary therapist and clinical provider extenders to assist with the twice-daily, brief feedback sessions that are held after the patient completes in vivo exposure exercises and listens to the PE session audio recording.	Having a therapy team allows for supplemental coverage in the absence or unavailability of the primary therapist and helps prevent having any missed sessions during the 3-week daily intensive outpatient program.
2. Clinic-Based Completion of Homework	Except for in vivo exposure exercises, participants complete all PE homework in the clinic.	The goal here is to help reduce avoidance, ensure the completion of the daily homework assignments, and assist with technical difficulties related to listening to the audio recording of the PE session.
3. Brief Therapist Feedback Sessions	Patients participate in 2 brief feedback sessions each day, once after completing the daily in vivo exposure and again after listening to the audio recording of their session.	The additional feedback provides added emotional support and opportunities for cognitive processing after exposure sessions, and it allows for immediate adjustment or titration of in vivo hierarchy.
4. Enhanced Social Support	A social-support person (e.g., spouse, partner, friend, etc.) is included in person or via telehealth during Session 2, as appropriate, for additional support during and after the treatment program.	Support systems sometimes inadvertently encourage avoidance behaviors. By involving a support person in education on the PE treatment rationale, common reactions to trauma, and importance of homework, that individual can better help foster rather than hinder recovery.
5. Top Three Traumas	The patient is provided the opportunity to process the top 3 traumas in imaginal exposure, as clinically indicated.	Many PTSD patients have experienced multiple traumas of different types. Addressing the top 3 traumas provides broader coverage for trauma processing.
6. Graduated Imaginal Exposure	Imaginal exposure is initiated with the least distressing of the top 3 trauma memories, followed by the second most distressing trauma, and finally with the index event.	Beginning imaginal exposure with the least distressing trauma memory is often easier and enhances mastery of the technique before addressing the second most distressing trauma and the index event.
7. Brief Timeline Review of All Traumas	After completing imaginal exposure for the most distressing trauma, the patient briefly reviews with the therapist all previous traumas that were not included in the top 3 traumas.	Imaginal exposure does not need to be conducted for every traumatic event in a person's life. This exercise shows that the skills learned in PE treatment can be used to address other traumas.
8. Posttreatment Booster Sessions	Three booster sessions (up to 1 hour in length) are provided at 1, 3, and 7 weeks posttreatment.	The extra sessions allow for a more tapered discontinuation of treatment after 3 intensive weeks of daily treatment and help patients generalize skills to the home environment.

© 2023 Peterson AL et al. *JAMA Network Open.*

eTable 2. Description of Study Measures

Measure	Description
**Demographics and Military Service Characteristics Form**	The Demographics and Military Service Characteristics Form is a self-report measure of standard demographics and information related to military service.
**Clinician Administered PTSD Scale for *DSM-5* (CAPS-5)**	The CAPS-5[1] is a structured diagnostic interview assessing for posttraumatic stress disorder (PTSD). Symptom severity over the past month is measured on a 5-point ordinal ranking scale. CAPS-5 total scores range from 0 to 80, with higher scores indicating greater symptom severity. The CAPS-5 was administered at baseline and at 1-, 3-, and 6-month follow-up. It has excellent psychometric properties in veteran samples, with high internal consistency (α = .88), good interrater reliability (kappa = .78), good test-retest reliability (ICC = .78), and convergent and discriminant validity.[1-3]
**PTSD Checklist for *DSM-5* (PCL-5)**	The PCL-5[4,5] is a 20-item, self-report measure of PTSD symptoms. Symptom severity is measured on a 5-point Likert scale in which participants report how bothered they were by their symptoms in the past month, from 0 = *not at all* to 4 = *extremely*. PCL-5 total scores range from 0 to 80, with higher scores indicating greater symptom severity. The PCL-5 was administered at baseline, weekly during treatment (interim assessments 1, 2, and 3), and at 1-, 3-, and 6-month follow-up assessments. It has demonstrated excellent psychometric properties in civilian and military samples, with high internal consistency for the total PCL-5 score (α = .91 to .96), good test-retest reliability (*r* = .82 to .84), and convergent and discriminant validity.[4-6]
**Sheehan Disability Scale (SDS)**	The SDS[7-9] is a 3-item, self-report measure of functional impairment due to mental health difficulties. Each item is rated on a 10-point visual analog scale. Global functional impairment scores range from 0 to 30, with higher scores indicating greater impairment. The SDS was administered at baseline and 1-, 3-, and 6-month follow-ups. It has excellent psychometric properties, with high internal consistency (α = .79 to .91), good test-retest reliability (ICC = .72 to .73) and demonstrated convergent validity.[10,11]
**Brief Inventory of Psychosocial Functioning (B-PIF)**	The B-PIF[12,13] is a 7-item, self-report instrument measuring respondents' level of functioning in 7 life domains: romantic relationship, relationship with children, family relationships, friendships and socializing, work, training and education, and activities of daily living.[13] Respondents indicate the degree to which they had trouble in the last 30 days in each area on a 7-point scale ranging from 0 = *Not at all* to 6 = *Very much*. The B-IPF was administered at baseline and 1-, 3-, and 6-month follow-ups. The B-IPF has demonstrated concurrent validity, and the full 80-item IPF from which it was created has strong test-retest reliability and internal consistency.[13]

© 2023 Peterson AL et al. *JAMA Network Open.*

eTable 3. Summary of Adverse Events (AEs) Reported During Treatment

**Measures**	**Total**	**IOP-PE**	**Massed-PE**	**# “Related”**
# Subjects randomized	234	117	117	234
# Subjects reporting AEs	49	25	24	36
% Subjects reporting AEs	20.9%	21.4%	20.5%	15.4%
# AEs reported	154	90	64	94
Mean # AEs/subjects reporting	3.1	3.6	2.7	2.6
# Serious AEs reported	3	2	1	1
**AEs Reported**				
Increased PTSD symptoms	7	6	1	6
Nightmare	10	7	3	10
Other sleep disturbance	12	8	4	10
Anxiety, worry	14	10	4	12
Panic	3	2	1	2
Paranoia	1	0	1	1
Depression, sadness	12	5	7	7
Guilt, shame	2	1	1	1
Anhedonia	2	1	1	0
Fatigue	4	2	2	3
Increased stress	7	3	4	4
Increased emotionality	2	0	2	0
Irritability	7	4	3	5
Anger	9	7	2	6
Frustration	3	2	1	1
Isolation	2	1	1	1
Marital strife	2	2	0	0
Back pain	4	4	0	1
Other pain	12	7	5	2
Muscle spasms	2	0	2	0
Migraine	3	2	1	1
Other headache	4	2	2	3
Injury	2	1	1	0
Cold symptoms	1	1	0	0
GI disturbance	5	0	5	0
Nausea	3	2	1	3
Vomiting	3	2	1	2
Palpitations	1	0	1	0
Dizziness	1	1	0	1
Blurred vision	1	0	1	0
Asthma	1	1	0	1
Shortness of breath	1	0	1	1
Decreased appetite	1	1	0	1
Weight loss	1	1	0	1
**Total AEs Reported**	**154**	**90**	**64**	**94**

Abbreviations: AE, adverse event; GI, gastro-intestinal; IOP-PE, Intensive Outpatient Program Prolonged Exposure; Massed-PE, Massed Prolonged Exposure.

© 2023 Peterson AL et al. *JAMA Network Open.*

eReferences

[1] Weathers FW, Bovin MJ, Lee DJ, et al. The Clinician-administered PTSD Scale for DSM–5 (CAPS-5): development and initial psychometric evaluation in military veterans. *Psychol Assess*. 2018;30(3):383-395. doi:10.1037/pas0000486

[2] Weathers FW, Ruscio AM, Keane TM. Psychometric properties of nine scoring rules for the Clinician-Administered Posttraumatic Stress Disorder Scale. *Psychol Assess*. 1999;11(2):124-133. doi:10.1037/1040-3590.11.2.124

[3] Weathers FW, Keane TM, Davidson JRT, Weathers FW, Keane TM, Davidson JR. Clinician-Administered PTSD Scale: a review of the first ten years of research. *Depress Anxiety*. 2001;13(3):132-156. doi:10.1002/da.1029

[4] Blevins CA, Weathers FW, Davis MT, Witte TK, Domino JL. The Posttraumatic Stress Disorder Checklist for DSM-5 (PCL-5): development and initial psychometric evaluation. *J Trauma Stress*. 2015;28(6):489-498. doi:10.1002/jts.22059

[5] Bovin MJ, Marx BP, Weathers FW, et al. Psychometric properties of the PTSD Checklist for Diagnostic and Statistical Manual of Mental Disorders–Fifth Edition (PCL-5) in veterans. *Psychol Assess*. 2016;28(11):1379-1391. doi:10.1037/pas0000254

[6] Wortmann JH, Jordan AH, Weathers FW, et al.; on behalf of the STRONG STAR Consortium. Psychometric analysis of the PTSD Checklist-5 (PCL-5) among treatment-seeking military service members. *Psychol Assess*. 2016;28(11):1392-1403. doi:10.1037/pas0000260

[7] Sheehan DV. *The Anxiety Disease*. Charles Scribner Sons; 1983.

[8] Sheehan DV, Harnett-Sheehan K, Raj BA. The measurement of disability. *Int Clin Psychopharmacol*. 1996;11(Suppl 3):89-95. doi:10.1097/00004850-199606003-00015

[9] Sheehan KH, Sheehan DV. Assessing treatment effects in clinical trials with the Discan metric of the Sheehan Disability Scale. *Int Clin Psychopharmacol*. 2008 ;23(2):70-83. doi:10.1097/YIC.0b013e3282f2b4d6

[10] Arbuckle R, Frye MA, Brecher M, et al. The psychometric validation of the Sheehan Disability Scale (SDS) in patients with bipolar disorder. *Psychiatry Res*. 2009;165(1-2) :163-174. doi:10.1016/j.psychres.2007.11.018

[11] Coles T, Coon C, DeMuro C, McLeod L, Gnanasakthy A. Psychometric evaluation of the Sheehan Disability Scale in adult patients with attention-deficit/hyperactivity disorder. *Neuropsychiatr Dis Treat*. 2014;10:887-895. doi:10.2147/NDT.S55220

[12] Rodriguez P, Holowka DW, Marx BP. Assessment of posttraumatic stress disorder-related functional impairment: a review. *J Rehabil Res Dev*. 2012;49(5):649-665. doi:10.1682/JRRD.2011.09.0162.

[13] Kleiman SE, Bovin MJ, Black SK, et al. Psychometric properties of a brief measure of posttraumatic stress disorder-related impairment: The Brief Inventory of Psychosocial Functioning. *Psychol Serv*. 2020;17(2):187-194. doi:10.1037/ser0000306

© 2023 Peterson AL et al. *JAMA Network Open.*
