# Supplementary material for: Massed vs Intensive Outpatient Prolonged Exposure for Combat-Related Posttraumatic Stress Disorder: A Randomized Clinical Trial
Source: JAMA Netw Open. 2023 Jan 5;6(1):e2249422. doi: 10.1001/jamanetworkopen.2022.49422 (PMC9856757; doi:10.1001/jamanetworkopen.2022.49422)
Supplement: Supplement 3. — Data Sharing Statement [file jamanetwopen-e2249422-s003.pdf]

Data Sharing Statement

Peterson. Massed vs Intensive Outpatient Prolonged Exposure for Combat-Related Posttraumatic Stress Disorder. *JAMA Netw Open*. Published January 05, 2023. doi:10.1001/jamanetworkopen.2022.49422

Data
Data available: Yes
Data types: Deidentified participant data
How to access data: repository@strongstar.org
When available: With publication

Supporting Documents
Document types: Informed consent form
How to access documents: repository@strongstar.org
When available: With publication

Additional Information
Who can access the data: Researchers whose proposed use of the data has been approved
Types of analyses: For a specified purpose
Mechanisms of data availability: With investigator support, after approval of a proposal, and with a signed data access agreement
Any additional restrictions: Agreement to not attempt to identify or contact research participants.
